# Supplementary material for: A Genetically Tractable, Natural Mouse Model of Cryptosporidiosis Offers Insights into Host Protective Immunity
Source: Cell Host Microbe. 2019 Jul 10;26(1):135–146.e5. doi: 10.1016/j.chom.2019.05.006 (PMC6617386; doi:10.1016/j.chom.2019.05.006)
Supplement: Document S1. Figures S1–S6, Tables S1 and S2 [file mmc1.pdf]

Cell Host & Microbe, Volume *26*

Supplemental Information

A Genetically Tractable, Natural Mouse Model of Cryptosporidiosis Offers Insights into Host Protective Immunity

Adam Sateriale, Jan Šlapeta, Rodrigo Baptista, Julie B. Engiles, Jodi A. Gullicksrud, Gillian T. Herbert, Carrie F. Brooks, Emily M. Kugler, Jessica C. Kissinger, Christopher A. Hunter, and Boris Striepen

Table S1: Comprehensive diagnostic panel for quarantined mice, related to Figure 1

Laboratory animal parasitology panel	Laboratory animal pathogen PCR diagnostics	Laboratory animal mite PCR	Laboratory animal pinworm PCR
Direct exam	Mouse parvovirus	*Myobia musculi*	*Aspicularis tetraptera*
Fecal float	*Mycoplasma pulomonis*	*Myocoptes musculinis*	*Syphacia muris*
Anal tape	Lymphocytic choriomeningitis		
Ectoparasite exam	Mouse Adenovirus		
	Ectromelia virus		
	Polyoma virus		
	Minute virus of mice		
	Mouse hepatitis virus		
	Clostridium piliforme		
	Theiler's encephalomyelitis virus		
	Mouse rotavirus		
	Sendai virus		
	Pneumonia virus of mice		
	Reovirus		

Table S2: Sequences of primers and probes used in this study, related to Star Methods

Purpose	Primer/probe name	Sequence (5′ to 3′)
qPCR detection of Cryptosporidium	18S-JVA-F1	ATGACGGGTAACGGGGAAT
	18S-JVA-R1	CCAATTACAAAACCAAAAAGTCC
	18S-JVA-probe	[FAM]CGCGCCTGCTGCCTTCCTTAGATG[BHQ1]
PCR diagnostics of genetically manipulated locus	TK5F1	TTCCTCTTCCTTATTATACCCACCTAA
	TK3R1	CAGCTTCTTCTCCACCTGATAATATAGTATCTGTACC
	NeoF1	CGTATGCCCGACGGTGAAGATCTTG
	ActinR1	CTATTATTACTGTTTATACCAACTCATTCTGAAG
PCR diagnostics of collected mouse fecal pellets	BtkF1	AATGGGCTAGCGTAGTGCAG
	BtkR1	AGGGGACGTACACTCAGCTTT
	Zfy2F1	CATTAAGACAGAAAAGAC
	Zfy2R1	GTGAGGAAATTTCTTCCT
Guide RNA sequence for Cas9	TKendF	GTTGGAAGAATATAATCTCTGAGG
	TKendR	AAACCCTCAGAGATTATATTCTTC

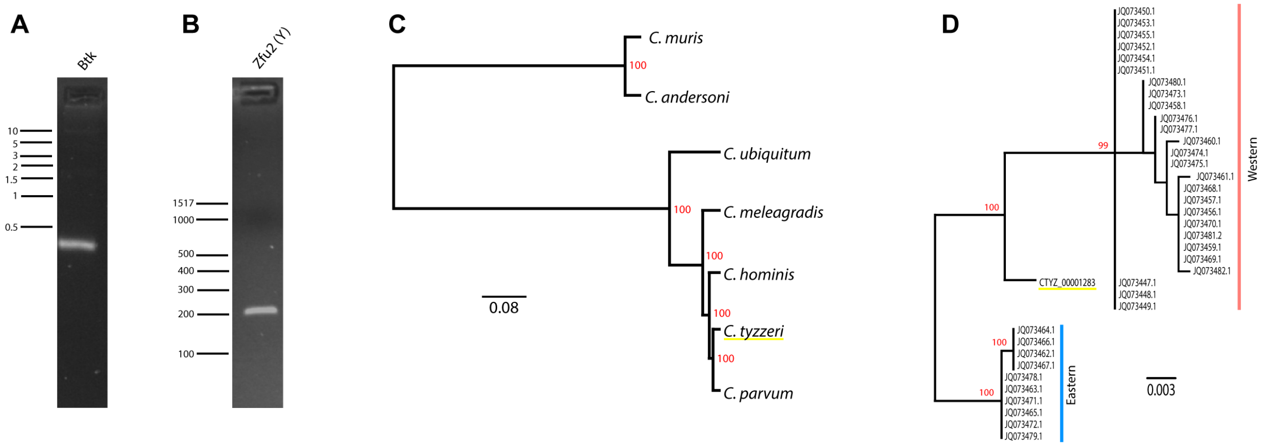

Figure S1: PCR and phylogenetic analysis, related to Figure 1 (A-B) PCR of DNA from field collected fecal pellets identifies *Mus musculus domestius* as natural *C. tyzzeri* host. **(A)** Gel of PCR using primers specific for a 342 bp segment of the Bruton agammaglobulinemia tyrosine kinase gene (Btk). This gene is present on the X chromosome of *Mus musculus domesticus*, but absent in *M. m. musculus*. **(B)** PCR using primers for the Zfu2 marker, which amplifies a 202 bp segment of both zinc finger protein 1 (Zfy1), and zinc finger protein 2 (Zfy2) on the Y chromosome. The genome of *M. m. musculus* has an 18 bp deletion in the last exon of Zfy2, producing two bands (202 and 184), whereas *M. m. domesticus* produces a single band (202). **(C-D)** Phylogenetic analysis of *Cryptosporidium tyzzeri.* **(C)** Whole genome phylogenetic tree constructed using 2764 single copy orthologous genes from *Cryptosporidium* species for which a full genome sequence data is available (available for download at CryptoDB.org). Single gene orthologs were identified using OrthoFinder (Emms and Kelly, 2015) and the tree was constructed using PhyML (Guindon et al., 2010) with JTT+1 settings and 1000 bootstraps for topology statistics. Genomes used for this analysis were: *Cryptosporidium muris* RN66, *Cryptosporidium andersoni* isolate 30847, *Cryptosporidium ubiquitum* isolate 39726, *Cryptosporidium meleagradis* strain UKMEL1, *Cryptosporidium hominis* 30976, *Cryptosporidium tyzzeri* UGA55, and *Cryptosporidium parvum* Iowa II. **(D)** Phylogenetic tree comparing gp60 sequences from *Cryptosporidium tyzzeri* UGA55 (CTYZ_00001283) and those PCR amplified from mice on the Eastern and Western side of the *M. m. musculus*–*M. m. domesticus* hybrid zone spanning from the Czech Republic to Germany see Kvac et al. (Kvac et al., 2013) for sequences and further detail. The alignments were constructed with MAFFT (Katoh and Standley, 2013), the phylogenetic tree was constructed with RaXML (Stamatakis, 2014) with 100 bootstrap replicates. Only branches with significant support are labeled. We note that using the current data set we direct lineage to either the Eastern or Western European *C. tyzzeri* cannot be assigned.

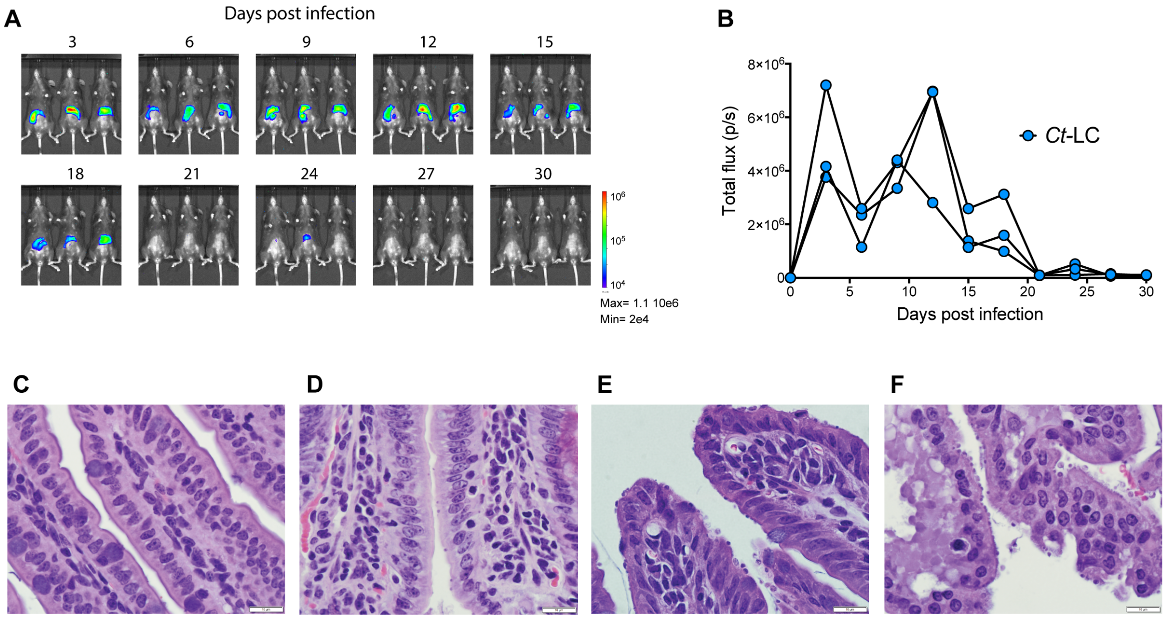

Figure S2: Whole animal imaging with Ct-LC parasites and histology of infected ileum sections, related to Figure 5 (A-B) Luminescence imaging of C57BL/6 mice infected with *Ct*-LC parasites. **(A)** Whole animal imaging of C57BL/6 mice infected with 10,000 *Ct*-LC oocysts as shown in Figure 3. **(B)** Parasite tissue burden represented as total flux (p/s) measurements. Each line represents a single mouse. **(C-F)** Histology of Ifn$\gamma^{-/-}$ and C57BL/6 mice. Hematoxylin and eosin stained sections of ilial tissue taken from mice on the tenth day of *Cryptosporidium* infection. Mice were 8 weeks old at the time of infection and were inoculated with 50,000 oocysts by gavage. **(C)** C57BL/6 mice infected with *Cryptosporidium parvum*, **(D)** C57BL/6 mice infected with *Cryptosporidium tyzzeri*, **(E)** Ifn$\gamma^{-/-}$ mice infected with *Cryptosporidium parvum*, and **(F)** Ifn$\gamma^{-/-}$ mice infected with *Cryptosporidium tyzzeri*.

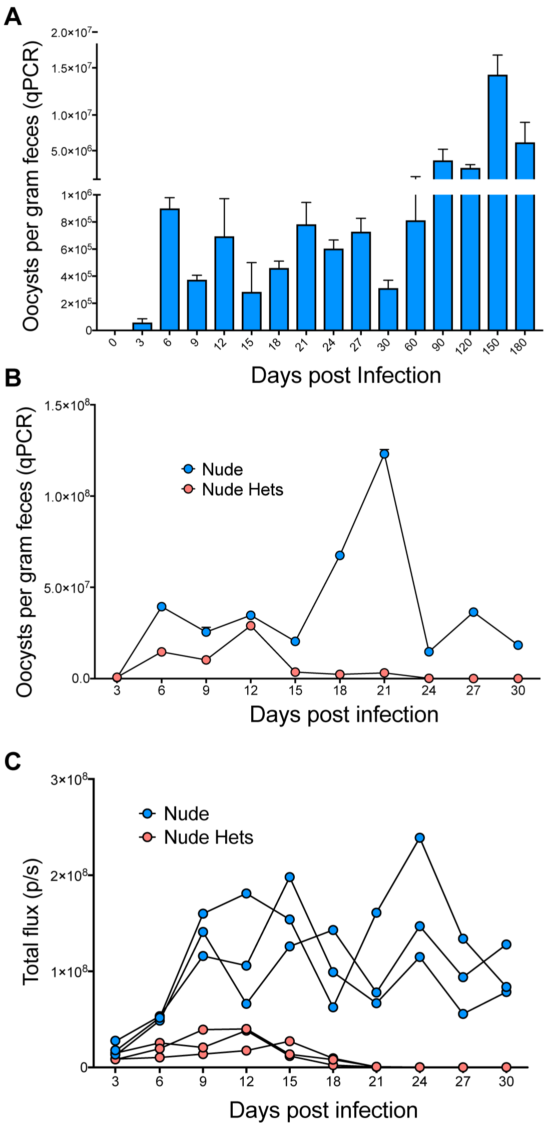

Figure S3: Infection in Rag$^{-/-}$ and athymic mice, related to Figure 5 (A) Extended time course of parasite burden in Rag$^{-/-}$ mice. Eight week old Rag1$^{-/-}$ mice infected with 50,000 *C. tyzzeri* oocysts each. Parasite shedding measured by qPCR from DNA isolated from pooled fecal samples. Two technical replicates and data shown is mean ± s.d., and *n* = 4 mice. **(B-C)** Fecal shedding and luminescence imaging of athymic mice. Foxn1^{nu} mice were infected with 10,000 transgenic *Ct*-LC oocysts. **(B)** Parasite shedding measured by qPCR from pooled fecal collections. Two technical replicates and data shown is mean ± s.d.. **(C)** Parasite tissue burden measured by whole animal imaging for individual mice. *n* = 6 mice total, 3 mice per group.

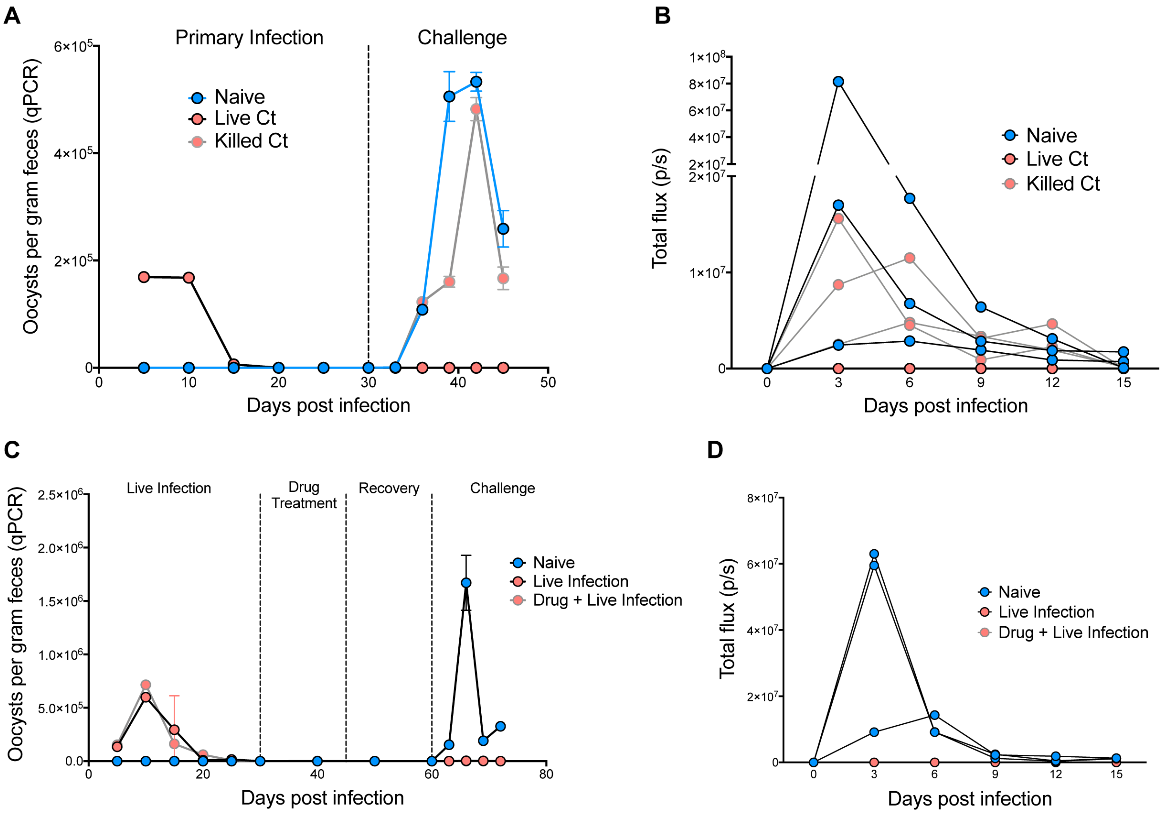

Figure S4: Fecal shedding and luminescent imaging of previously infected mice, related to Figure 6 (A-B) C57BL/6 mice were subjected to primary infection as indicated, followed by a challenge with 100,000 oocysts of transgenic *tyzzeri Ct*-LC. **(A)** Parasite shedding measured by qPCR of collected fecal samples throughout the primary infection and challenge. Two technical replicates and data shown is mean ± s.d.. **(B)** Parasite tissue burden measured by whole animal imaging following challenge. *n* = 9 mice, 3 per group. **(C-D)** Fecal shedding and luminescent imaging of drug treated mice. C57BL/6 mice were first subjected to primary infection with 10,000 live, wild-type, *C. tyzzeri* oocysts. Mice were then treated at day 30 with paromomycin to eradicate parasite infection (16mg/mL ad libitum) for 15 days and allowed to recover for an addition 15 days. At day 60 post infection, mice were challenged with 100,000 transgenic *Ct*-LC oocysts. **(C)** Parasite shedding measured by qPCR of collected fecal samples throughout the experiment. Two technical replicates and data shown is mean ± s.d.. **(D)** Parasite tissue burden measured by whole animal imaging following challenge. *n* = 9 mice, 3 per group.

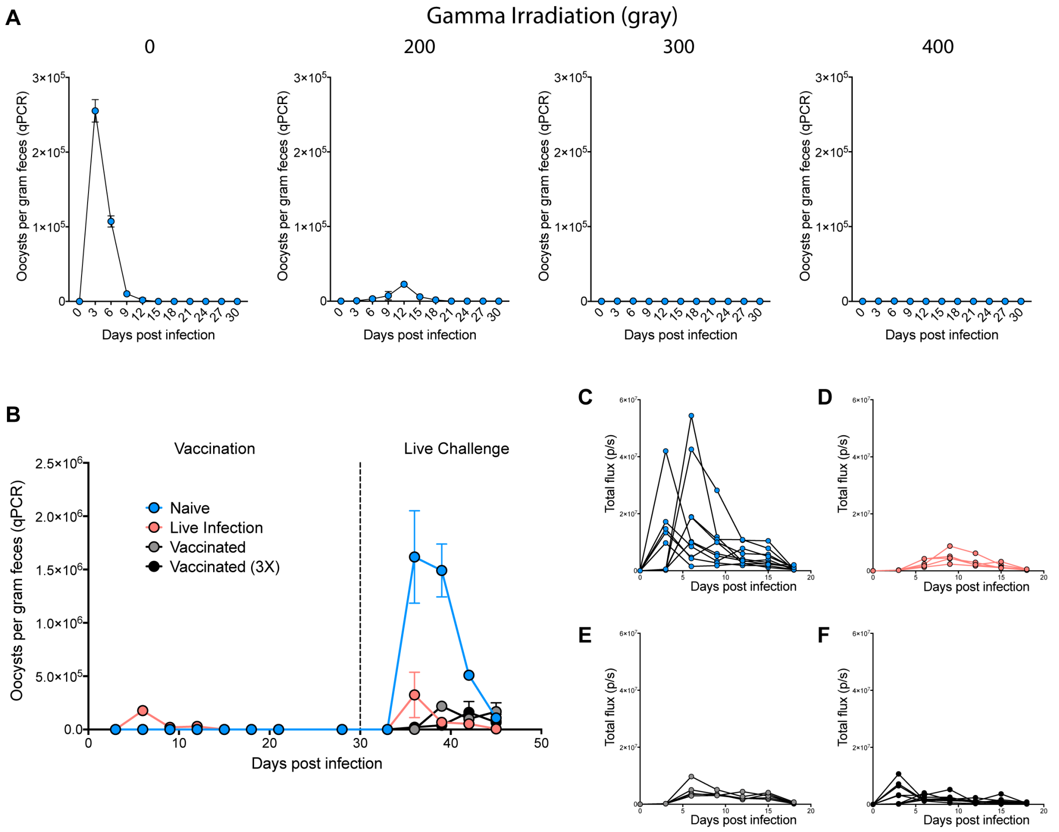

Figure S5: Attenuation of *Cryptosporidium tyzzeri* oocysts and fecal shedding and luminescent imaging of vaccinated mice, related to Figure 6 (A) Wild-type *C. tyzzeri* oocysts were exposed to the indicated dose of γ-irradiation using a Cs-137 or mock treatment, then mice were infected with 100,000 oocysts each and fecal shedding was monitored by qPCR. *n* = 12 mice, 3 per group, with qPCR performed on pooled fecal material with two technical replicates (data shown is mean ± s.d.). **(B-F)** Fecal shedding and luminescence imaging of vaccinated mice. C57BL/6 mice were first infected with 50,000 live or gamma irradiation attenuated (300gray) wild-type *C. tyzzeri* oocysts, then challenged with 100,000 live, transgenic, *Ct*-LC oocysts. **(B)** Parasite shedding measured by qPCR of collected fecal samples throughout the infection/vaccination and subsequent live challenge (data shown is mean ± s.d.). **(C-F)** Parasite tissue burden measured by whole animal imaging following the live challenge. *n* = 30 mice across 2 independent experiments, 10 Naïve, 5 Live Infection, 5 Vaccinated, and 10 Vaccinated (3X).

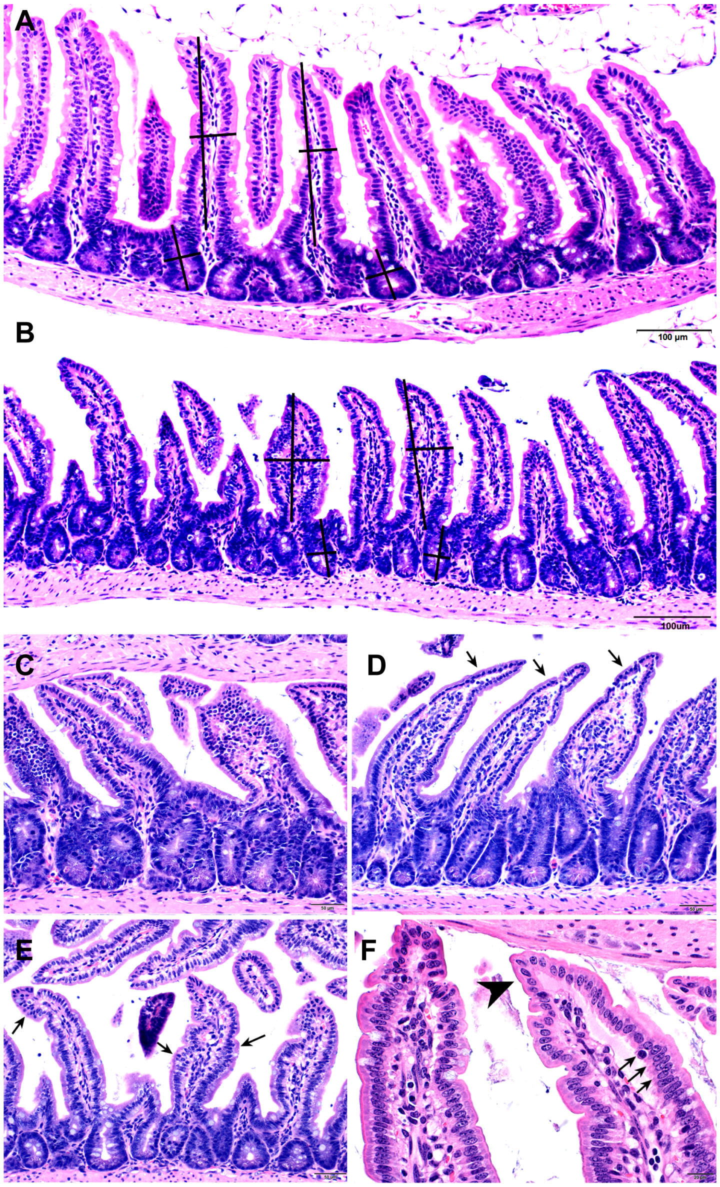

Figure S6: Select features of histologic scoring system, related to Star Methods and Figure 3 **(A-B)**, 10X magnification, H&E stained photomicrographs of distal jejunal segments from uninfected **(A)** and day 6 infected **(B)** mice depicting unlabeled measurement bars for villus height, mid-villus width, crypt height, and crypt width; scale bars = 100 µm. **(C-E)** 20X magnification, H&E stained photomicrographs of distal jejunal segments from day 6 infected mice; scale bars = 50 µm. **(C)** Greater than 2 crypt branches with distorted lumens affect multiple crypts within the section. Crypts also have an increased mitotic index when compared to uninfected mice. **(D)** Attenuated (cuboidal), hyperbasophilic epithelium having high nuclear:cytoplasmic ratios (**arrows**) line mid- to apical portions of multiple villi. **(E)** Villus epithelial dysplasia characterized by malignment of villus epithelial nuclei with cell piling (arrows). **(F)** 40X magnification, H&E stained photomicrograph. The villus to the right is lined

attenuated epithelium (**arrowhead**) with a cleft (**arrows**) filled with pale eosinophilic fluid separating the basal aspects of epithelium from the lamina propria; scale bar = 20 µm.
