# Supplementary material for: A Genetically Tractable, Natural Mouse Model of Cryptosporidiosis Offers Insights into Host Protective Immunity
Source: Cell Host Microbe. 2019 Jul 10;26(1):135–146.e5. doi: 10.1016/j.chom.2019.05.006 (PMC6617386; doi:10.1016/j.chom.2019.05.006)
Supplement: Document S2. Article plus Supplemental Information [file mmc2.pdf]

Resource

Cell Host & Microbe

A Genetically Tractable, Natural Mouse Model of Cryptosporidiosis Offers Insights into Host Protective Immunity

Graphical Abstract

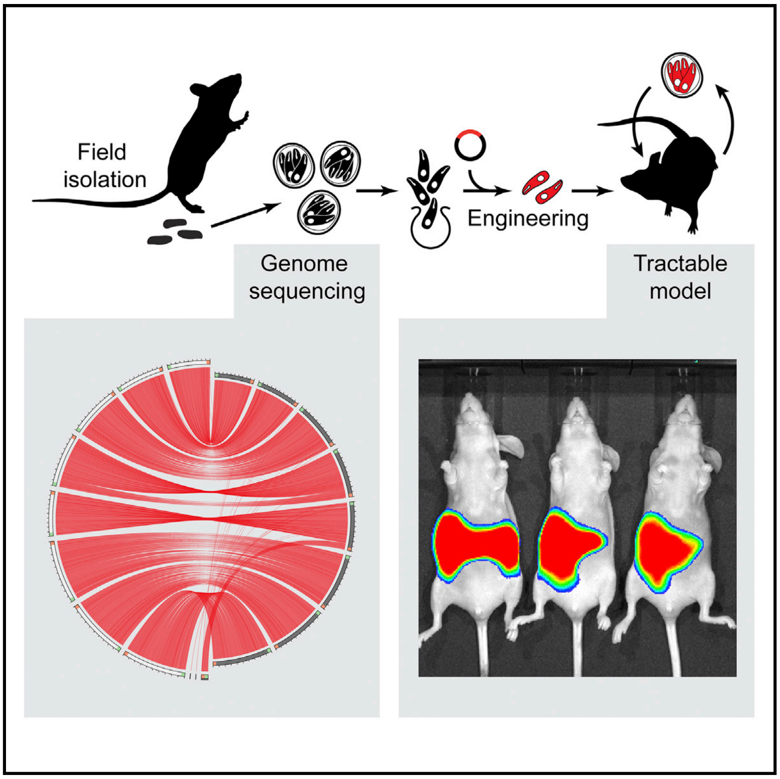

Authors

Adam Sateriale, Jan Šlapeta, Rodrigo Baptista, ..., Jessica C. Kissinger, Christopher A. Hunter, Boris Striepen

Correspondence

striepen@upenn.edu

In Brief

Cryptosporidiosis is an important diarrheal disease that lacks effective treatment and vaccination. Sateriale et al. isolate *Cryptosporidium tyzzeri* from naturally infected wild mice and derive a model of infection in immunocompetent mice in which both parasite and host are genetically tractable. The mechanisms of protective immunity are now open to analysis.

Highlights

- We isolated and sequenced *Cryptosporidium tyzzeri*, a natural mouse pathogen
- *C. tyzzeri* can be genetically manipulated using CRISPR-driven homologous repair
- *C. tyzzeri* models human cryptosporidiosis with T cell- and IFN-γ-dependent resolution
- Mice develop protective immunity following both live infection and vaccination

Sateriale et al., 2019, Cell Host & Microbe *26*, 135–146
July 10, 2019 © 2019 The Authors. Published by Elsevier Inc.
https://doi.org/10.1016/j.chom.2019.05.006

A Genetically Tractable, Natural Mouse Model of Cryptosporidiosis Offers Insights into Host Protective Immunity

Adam Sateriale,[1] Jan Šlapeta,[2] Rodrigo Baptista,[3] Julie B. Engiles,[1] Jodi A. Gullicksrud,[1] Gillian T. Herbert,[3] Carrie F. Brooks,[3] Emily M. Kugler,[1] Jessica C. Kissinger,[3,4,5] Christopher A. Hunter,[1] and Boris Striepen[1,6,*]

[1]Department of Pathobiology, School of Veterinary Medicine, University of Pennsylvania, 380 South University Avenue, Philadelphia, PA 19104, USA

[2]Sydney School of Veterinary Science, Faculty of Science, University of Sydney, Sydney, NSW 2006, Australia

[3]Center for Tropical and Emerging Global Diseases, University of Georgia, Paul D. Coverdell Center, 500 D.W. Brooks Drive, Athens, GA 30602, USA

[4]Department of Genetics, University of Georgia, Paul D. Coverdell Center, 500 D.W. Brooks Drive, Athens, GA 30602, USA

[5]Institute of Bioinformatics, University of Georgia, Paul D. Coverdell Center, 500 D.W. Brooks Drive, Athens, GA 30602, USA

[6]Lead Contact

*Correspondence: striepen@upenn.edu

https://doi.org/10.1016/j.chom.2019.05.006

SUMMARY

Cryptosporidium is a leading cause of diarrheal disease and an important contributor to early childhood mortality, malnutrition, and growth faltering. Older children in high endemicity regions appear resistant to infection, while previously unexposed adults remain susceptible. Experimental studies in humans and animals support the development of disease resistance, but we do not understand the mechanisms that underlie protective immunity to *Cryptosporidium*. Here, we derive an *in vivo* model of *Cryptosporidium* infection in immunocompetent C57BL/6 mice by isolating parasites from naturally infected wild mice. Similar to human cryptosporidiosis, this infection causes intestinal pathology, and interferon-γ controls early infection while T cells are critical for clearance. Importantly, mice that controlled a live infection were resistant to secondary challenge and vaccination with attenuated parasites provided protection equal to live infection. Both parasite and host are genetically tractable and this *in vivo* model will facilitate mechanistic investigation and rational vaccine design.

INTRODUCTION

Cryptosporidiosis, initially recognized as an opportunistic infection associated with advanced HIV-AIDS, is now known as a leading cause of diarrheal disease in immunocompetent individuals around the world (Checkley et al., 2015; Striepen, 2013). Young children are particularly vulnerable to infection with the apicomplexan parasite *Cryptosporidium*. In the Global Enteric Multicenter Study, the largest study of its kind to assess the etiology of early childhood diarrheal disease in sub-Saharan Africa and southeast Asia, *Cryptosporidium* was strongly associated with moderate-to-severe diarrhea and death across all study sites (Kotloff et al., 2013). Children that survive these life-threatening *Cryptosporidium* infections remain prone to malnutrition and stunted growth, with measurable decreases in height-for-age scores (Checkley et al., 1998; Mølbak et al., 1997; Mondal et al., 2009; Platts-Mills et al., 2015). A recent meta-analysis that accounted for growth faltering estimated over 12 million disability adjusted life years attributable to cryptosporidiosis in 2016 alone (Khalil et al., 2018). This massive impact on public health is exacerbated by the lack of tools to manage the disease; the current treatment for *Cryptosporidium* is of limited efficacy and new more potent medicines are still under development (Huston et al., 2015; Manjunatha et al., 2017) and no vaccine is available to prevent the infection.

Cryptosporidiosis in humans is predominantly caused by *Cryptosporidium hominis* and *Cryptosporidium parvum*. Epidemiology in children, as well as studies in human volunteers and in different animal species, have shown the development of immunity and suggest that vaccination may prevent the disease (Mead, 2010; Okhuysen et al., 1998), but the lack of animal models has limited vaccine development. Gnotobiotic piglets are susceptible to *C. hominis* and calves to *C. parvum*, but large animal models require specialized facilities (Tzipori, 1998; Widmer et al., 2000). Experiments in immunocompetent mice have been performed with *Cryptosporidium muris*; however, this species differs significantly from those infecting humans: phylogenetically, biochemically, and in the anatomical site of infection (stomach versus the small intestine). Mice are naturally resistant to *C. hominis* and *C. parvum*, but can be rendered susceptible to the latter by chemical immune suppression (Petry et al., 1995) or genetic immune insufficiency (Griffiths et al., 1998; Mead et al., 1991). These models have been critical to our understanding of innate mechanisms of resistance to *Cryptosporidium* and affirmed the important role of T cells in parasite control (Borad and Ward, 2010; Mead, 2014). However, because these studies are performed in neonates, immunocompromised or naturally resistant mice, we know little about the mechanisms required for long-lived protection.

© 2019 The Authors. Published by Elsevier Inc.
This is an open access article under the CC BY license (http://creativecommons.org/licenses/by/4.0/).

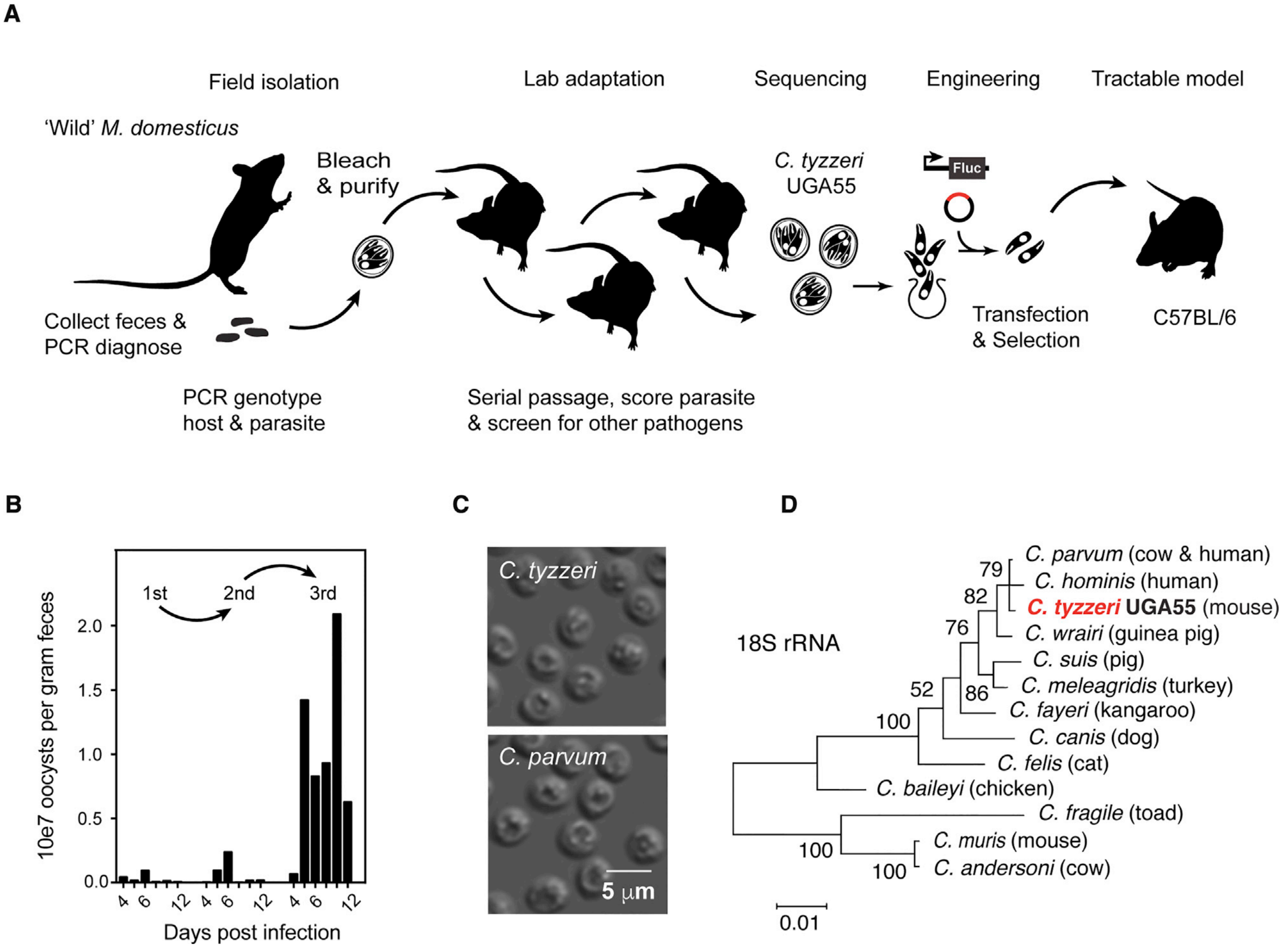

Figure 1. A Natural Mouse Model for Cryptosporidiosis

(A) Schematic overview of isolation and characterization of *Cryptosporidium tyzzeri*.

(B) Fecal shedding of *C. tyzzeri* in quarantined mice. Oocysts were purified from field-collected mouse feces and used to infect C57BL/6 mice, followed by serial passage. Fecal parasite burden is measured by qPCR.

(C) Differential interference contrast microscopy of *C. tyzzeri* and *C. parvum* oocysts. Note that *C. tyzzeri* oocysts isolated in this study are of similar shape and size as *C. parvum*.

(D) Maximum likelihood phylogenetic tree of the 18s rRNA locus of different *Cryptosporidium* species (see Figure S1C for a phylogenetic tree constructed with full genome alignments).

Robust rodent models with natural progression that replicate human disease have proven transformative for many fields of infection biology (Martina et al., 2003; Ploss et al., 2009). In order to develop such a model for cryptosporidiosis, we isolated a species of *Cryptosporidium* that naturally colonizes the mouse small intestine. This parasite, *Cryptosporidium tyzzeri*, genetically resembles those that cause human cryptosporidiosis and causes similar disease pathology. We then use CRISPR-driven genome engineering to create stable transgenic *C. tyzzeri* that express luminescent and fluorescent reporters. With these transgenic parasites we demonstrate acquired resistance to infection in mice and importantly such resistance can be elicited through vaccination with an attenuated parasite. Moving forward this fully tractable mouse model of cryptosporidiosis will permit rigorous investigation of potential vaccination strategies and allow the dissection of the complex host-pathogen interactions of *Cryptosporidium* infection.

RESULTS

Isolation of *Cryptosporidium tyzzeri* and *De Novo* Genome Assembly

Mouse fecal pellets were collected from farms in Athens, Georgia in the United States; PCR analyses showed the presence of DNA from mice of the species *Mus musculus domesticus* (Figures S1A and S1B), and for 30% of the samples, *Cryptosporidium* DNA. Oocysts were purified from feces, bleach treated, and then used to infect adult C57BL/6 mice, housed in quarantine, by gavage (Figure 1A). Cryptosporidium burden was monitored by measuring fecal parasite shedding by qPCR (Figure 1B) and mice were subjected to a panel of diagnostic tests and veterinary pathology to rule out co-infection with other pathogens (Table S1). Parasites were passaged three times in this fashion in isolation, with oocyst yield increasing in each passage and *Cryptosporidium* remained the sole pathogen detected.

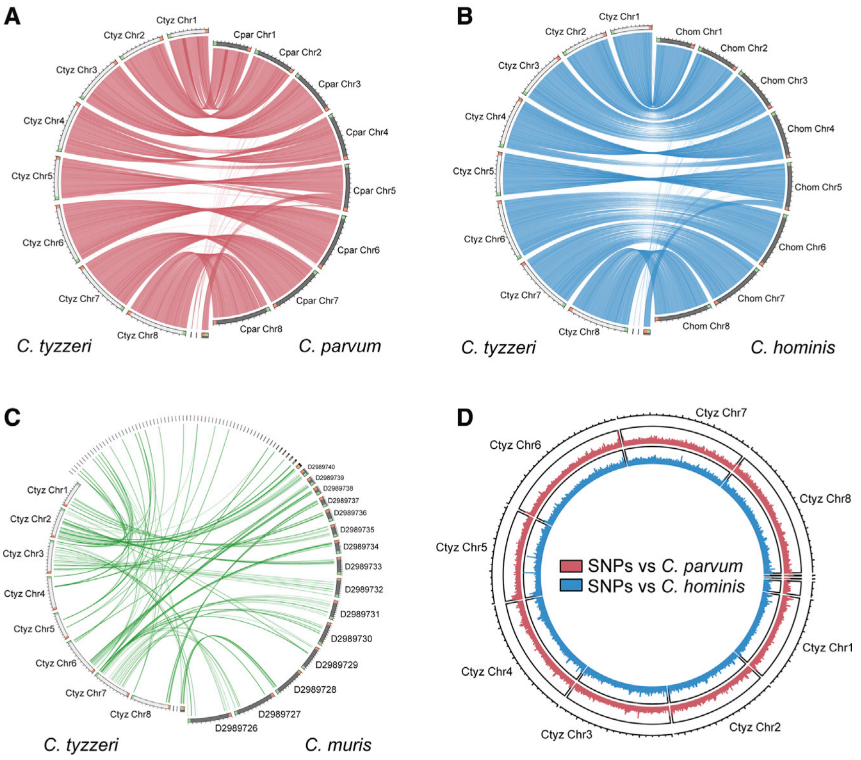

Figure 2. The Genome of *Cryptosporidium tyzzeri* Is Very Similar to the Genomes of *C. parvum* and *C. hominis*

(A–C) Synteny maps comparing the assembled genomic contigs of *C. tyzzeri* UGA55 and *C. parvum* UKP6 (A), *C. hominis* 30976 (B), and *C. muris* RN66 (C). Each line connects a genomic segment with a local alignment e-value cut-off of $1e^{-20}$. This correlates to >90% overall nucleotide identity.

(D) Single-nucleotide polymorphism (SNP) density map comparing *C. tyzzeri* with *C. parvum* (red) and *hominis* (blue) with a 1 kb rolling window.

Morphologically, oocysts from the current isolated strain, designated UGA55, are similar to those of *C. parvum* ([4.84 + 0.16] × [4.13 + 0.14] μm, Figure 1C) and distinct from the larger and more oval shaped *C. muris* oocysts. Genetically, sequencing of the 18S ribosomal RNA locus revealed a match to *C. tyzzeri*, a natural parasite of house mice (Ren et al., 2012; Kváč et al., 2013). The genome of *C. tyzzeri* isolate UGA55 was sequenced, assembled *de novo*, and manually annotated. This is the first assembled genome sequence for *C. tyzzeri*, which was made publicly available through GenBank and the *Cryptosporidium* Genomic Resource, CryptoDB (Heiges et al., 2006). Phylogenetic analyses place *C. tyzzeri* UGA55 in close proximity to the most prevalent human pathogens, *C. parvum* and *C. hominis*. This holds true regardless of whether this analysis is focused on 18S rRNA (Figure 1D) or expanded to the whole genome by using 2764 single-copy orthologous genes shared among the seven *Cryptosporidium* species for which full genome sequences are available (Figure S1C). When compared to the genomes of *C. parvum* and *C. hominis*, the genome of *C. tyzzeri* is remarkably similar in size, structure, and sequence. The genome of *C. tyzzeri* is distinct from *C. muris* as illustrated by the pronounced difference in the ability to map the genome sequences to each other in synteny plots when using a local alignment e-value cut-off of 1e-20 (Figure 2). There are 3977 predicted protein encoding genes in a highly AT-rich 9.02-Mb genome, with 95%–96% sequence identity at the nucleotide level to *C. hominis* and *C. parvum* (Figure 2; see Table 1 for a more thorough comparison). We identified 181,187 single-nucleotide polymorphisms relative to *C. parvum* and 223,396 for *C. hominis*, which are most abundant in telomeric regions that carry genes for highly variable secretory proteins (Abrahamsen et al., 2004; Xu et al., 2004). Note that in Figure 2D, SNP abundance peaks between *C. tyzzeri* and *C parvum* correlate with those between *C. tyzzeri* and *C. hominis*, and they also correlate with such peaks between *C. parvum* and *C. hominis* (data not shown).

Pathology of *Cryptosporidium tyzzeri* Infection in Mice Closely Resembles Human Disease

In C57BL/6 mice infected with *C. tyzzeri* parasites detected by qPCR were found throughout the small intestine with the highest burden in the ileum (Figure 3A) and they were absent from the stomach and the colon. Histological analysis of the small intestine during a time course of infection revealed pronounced pathological changes. Infected tissue showed a marked loss of the glycocalyx of the epithelial cells, effacement and blunting of villi, and a significant influx of leukocytes (see STAR Methods for the detailed scoring rubric that was developed). Within the crypts of the small intestine there was a significant increase in mitoses upon infection with *C. tyzzeri*, with crypt hyperplasia and increased crypt branching (Figures 3B–3H). Similar pathology has been reported from infected humans (Lumadue et al., 1998) and the associated histological and physiological changes have been linked to malabsorption and environmental enteropathy in children as a cause of growth stunting (Checkley et al., 1998; Khalil et al., 2018).

C. tyzzeri Is Genetically Tractable Using a CRISPR/Cas9 Approach

To make *C. tyzzeri* tractable for immunological studies we genetically engineered reporter parasites that can be detected *in vivo* to allow for precise longitudinal measurements of parasite burden. We harnessed a CRISPR/Cas9-driven homologous recombination strategy (Vinayak et al., 2015) to introduce a dual reporter and drug selection cassette into the thymidine kinase locus (Figure 4A). Transgenic parasites were then selected and propagated *in vivo* using paromomycin and PCR mapping demonstrated successful integration of the cassette into the *C. tyzzeri* genome at the desired locus (Figure 4B). Infection with these transgenic *C. tyzzeri* expressing red-shifted luciferase (strain Ct-LC) is readily detected by whole animal imaging upon injection of luciferin (Figure 4C) and can be seen in the small

Table 1. Comparative Analysis of *Cryptosporidium* Genomes

	C. tyzzeri	*C. hominis*	*C. parvum*	*C. muris*
Genome size (Mb)	9,015,713	9,059,225	9,102,324	9,245,251
Number of scaffolds	11	53	8	84
GC content (%)	30.25	30.13	30.22	28.47
Percent coding (%)	77.96	81.86	81.09	75.23
Gene density (genes per 100 Kb)	44.13	43.59	43.31	42.55
Total predicted protein encoding genes	3,977	3,949	3,941	3,934
Genes with introns (%)	15.31	14.84	14.59	20.36
Average gene length (bp)	1,736	1,852	1,834	1,747
Predicted proteins with:				
signal peptide	698	678	716	603
transmembrane domain	865	851	870	836
functional domain (interpro)	2,315	2,317	2,311	2,428
EC number	918	915	908	945
In comparison to *C. tyzzeri*:				
% genome aligned with >75% identity	-	99.2	99	7.2
Total SNPs within aligned region	-	223,396	181,187	18,054

intestine (Figure 4D) but not the stomach or colon. The strain also expresses the fluorescent protein reporter mCherry, allowing for parasite visualization within the infected tissue and cells (Figure 4E). It is also possible to dissociate sections of the small intestine into individual cells (see STAR Methods for detail) and to exploit the fluorescence of these transgenic parasites to specifically identify infected epithelial cells by flow cytometry (Figure 4F). Previous *in vitro* studies suggested that *Cryptosporidium* might interfere with IFNγ induced expression of the major histocompatibility complex (MHC) (Choudhry et al., 2009), but infection of mice led to global upregulation of epithelial cell MHC class I and II expression (data not shown). Taking advantage of the ability to distinguish infected and uninfected cells, we compared MHC expression of these populations. Uninfected (cherry negative) epithelial cells from the small intestine expressed high levels of MHC class I and II (Figure 4G). However, infected epithelial cells showed reduced MHC class I and II surface expression; these data provide *in vivo* evidence that *Cryptosporidium* may modulate or suppress aspects of immune detection within its natural host.

Resolution of *C. tyzzeri* Infection Is Dependent on Interferon Gamma and T Cells

Next, we explored the role of host immune function in colonization and clearance in normal and immunocompromised mice. When immunocompetent, adult C57BL/6 mice were challenged with *C. parvum*, the infection was undetectable by *in vivo* imaging and fecal parasite shedding (Figures 5A and 5B). Note that this was a viable and virulent inoculum that readily produced a patent infection in mice that lacked the gene for IFN-γ (Figure 5C). In contrast, challenge of C57BL/6 mice with the same dose of *C. tyzzeri* resulted in robust infection by either measurement (Figures 5A–5C). C57BL/6 mice controlled *C. tyzzeri* infection within 2–3 weeks, after which parasites were no longer detectable by whole animal imaging or fecal qPCR (Figures 5D, S2A, and S2B). We note that in healthy human adults, cryptosporidiosis is a self-limiting infection and disease that resolves with similar kinetics to those observed here for *C. tyzzeri* in mice (Jokipii and Jokipii, 1986).

While *C. tyzzeri* produces robust infection in wild-type mice, this infection is significantly exacerbated in *Ifn*$\gamma^{-/-}$ mice. During the first two weeks of infection, parasite burden in *Ifn*$\gamma^{-/-}$ mice infected with *C. tyzzeri* was approximately 100-fold higher than that of wild-type mice (Figure 5D). *C. tyzzeri* infection of *Ifn*$\gamma^{-/-}$ mice produces enhanced tissue burden and pathology observed by histological analysis of intestinal sections (Figures S2C–S2F) and *Ifn*$\gamma^{-/-}$ mice infected with more than 10,000 oocysts succumbed to the infection. While at lower doses there was eventual control of parasite numbers in *Ifn*$\gamma^{-/-}$ mice, we noted a failure to fully eradicate this infection. *Rag1*$^{-/-}$ mice, which lack functional B and T cells, show a similar early parasite burden to wild-type controls, yet fail to control *C. tyzzeri* and remain chronically infected. We monitored infection of *Rag1*$^{-/-}$ mice for 6 months and did not observe resolution over this time (Figure S3A). To assess the individual contribution of B and T cells, μMt^{-} mice that lack mature B cells and homozygous Foxn1^{nu} mice that lack thymus derived T cells were infected. The ability of μMt^{-} mice to control *C. tyzzeri* was comparable to wild-type mice, whereas homozygous Foxn1^{nu} mice remained chronically infected (Figures 5E, S3B, and S3C). Together this data demonstrates that T cells are critical for control in this mouse model, mirroring what is seen in humans. Indeed, cryptosporidiosis is a common opportunistic infection in HIV-AIDS patients, as well as immunosuppressed organ transplant recipients, causing severe and chronic disease in those with low numbers of CD4 T cells (Manabe et al., 1998; Flanigan et al., 1992; Schmidt et al., 2001). Overall, this comparison highlights the IFNγ dependent component of host specificity in *Cryptosporidium* and illustrates the importance of IFNγ for early and late resistance to *C. tyzzeri*. In addition, similar to what has been observed in HIV-AIDS patients (Manabe et al., 1998), T cells and not B cells have a critical role for clearance.

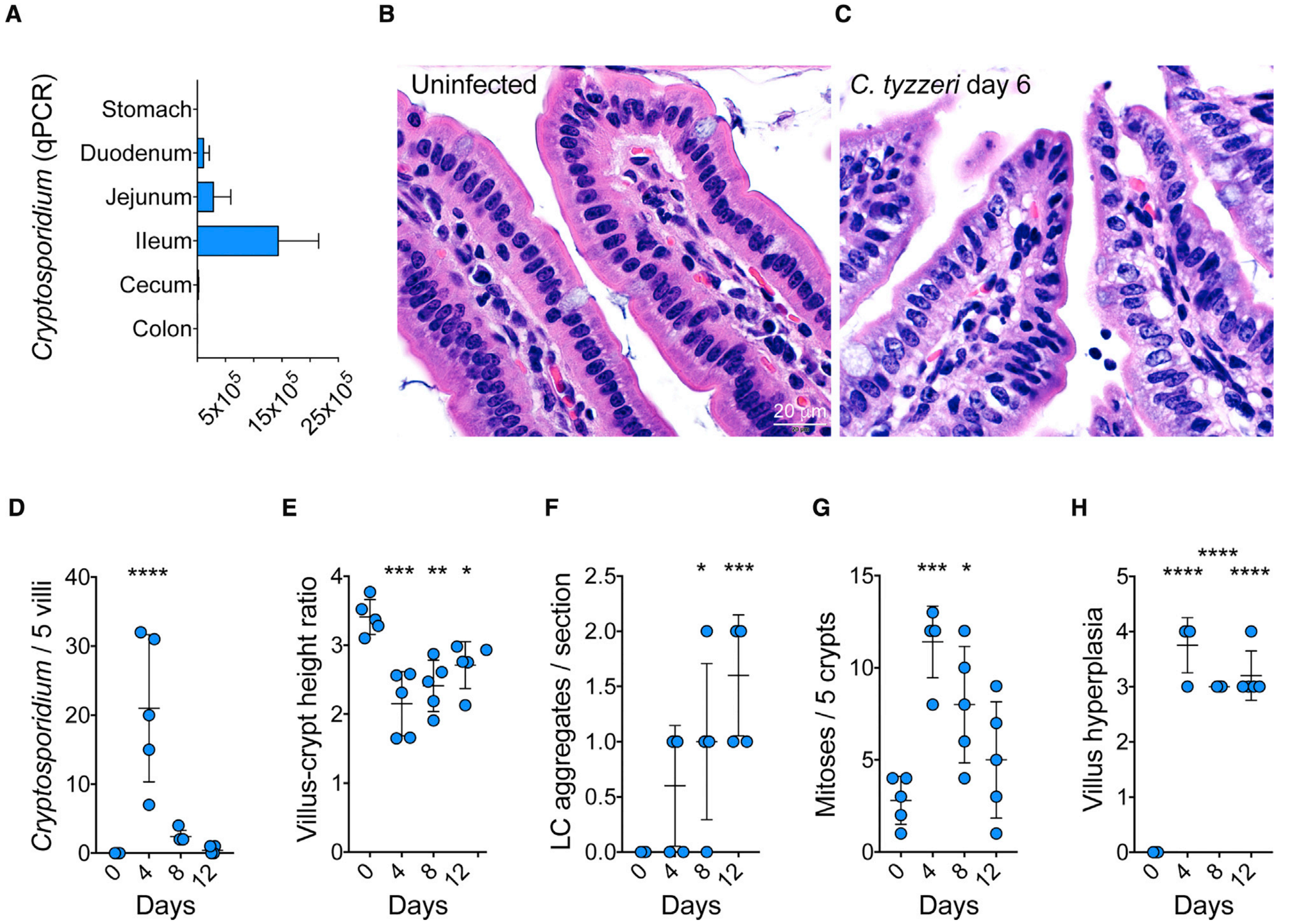

Figure 3. *Cryptosporidium tyzzeri* Infects the Small Intestine and Causes Pathology

(A) The intestinal tract from infected mice was resected and 1-cm^2 segments were tested for the presence of parasite DNA by qPCR. n = 7 mice from 2 separate experiments with tissue harvested at day 10 post infection with 10,000 oocysts.

(B and C) Hematoxylin and eosin stained sections of the distal small intestine of mice. Note tall columnar villus epithelium and intact apical microvilli border with associated glycocalyx in uninfected (B) mice, in contrast to severe villus attenuation, loss of the apical glycocalyx and effacement of microvilli in infected (C) animals.

(D–H) Mice were infected with 50,000 *C. tyzzeri* oocysts, killed at 0, 4, 8, and 12 days post infection and histologically scored for pathological changes over the course of infection. In the small intestine of infected mice, parasite burden peaked on day 4 post infection (D). Infection resulted in a marked reduction in villus:crypt height ratio (villus blunting) (E), lymphocyte aggregation (F), epithelial dysplasia with increased mitoses (G), and villus hyperplasia (H). n = 5 mice per group, with each dot representing the score for a single mouse, data are mean ± standard deviation (SD). Significance is determined using one-way ANOVA with Dunnett multiple comparison test (*p < 0.05, **p < 0.005, ***p < 0.0005, and ****p < 0.00005).

Primary Infection and Vaccination Induce Resistance to Subsequent Challenge

Children in endemic areas acquire resistance to cryptosporidiosis and while a single infection typically does not produce sterile immunity, it can prevent or lessen disease (Checkley et al., 2015; Okhuysen et al., 1998; Korpe et al., 2018). To test if this resistance can be modeled in mice, we assessed whether primary infection protects from secondary challenge (see schematic outline in Figure 6A). C57BL/6 mice were inoculated with live or heat-killed *C. tyzzeri* oocysts and infection was monitored by qPCR and by day-15 mice had cleared the infection (Figure S4A). Note that the parasites used in this primary infection were not marked by a genetic reporter. After 4 weeks, mice were challenged with transgenic *C. tyzzeri* expressing red-shifted luciferase and this secondary challenge was monitored by imaging (Figure S4B). In these experiments, mice that received heat-killed *C. tyzzeri* oocysts were not protected and had a parasite burden comparable to naive mice. However, mice that experienced a live *C. tyzzeri* infection demonstrated significantly lower infection upon secondary challenge (Figures 6B and 6C shows a summary of multiple experiments, p < 0.0001). For some intracellular pathogens, resistance to secondary challenge is dependent on concomitant immunity, which is lost with clearance of the initial infection (Belkaid et al., 2002). To assess whether a cryptic infection contributed to the resistance here, mice were treated for 2 weeks with paromomycin to eradicate potential persisting parasites (see Figure 6A in brackets). Even after this drug treatment, previously infected mice were resistant to secondary challenge (Figures 6D, S4C, and S4D).

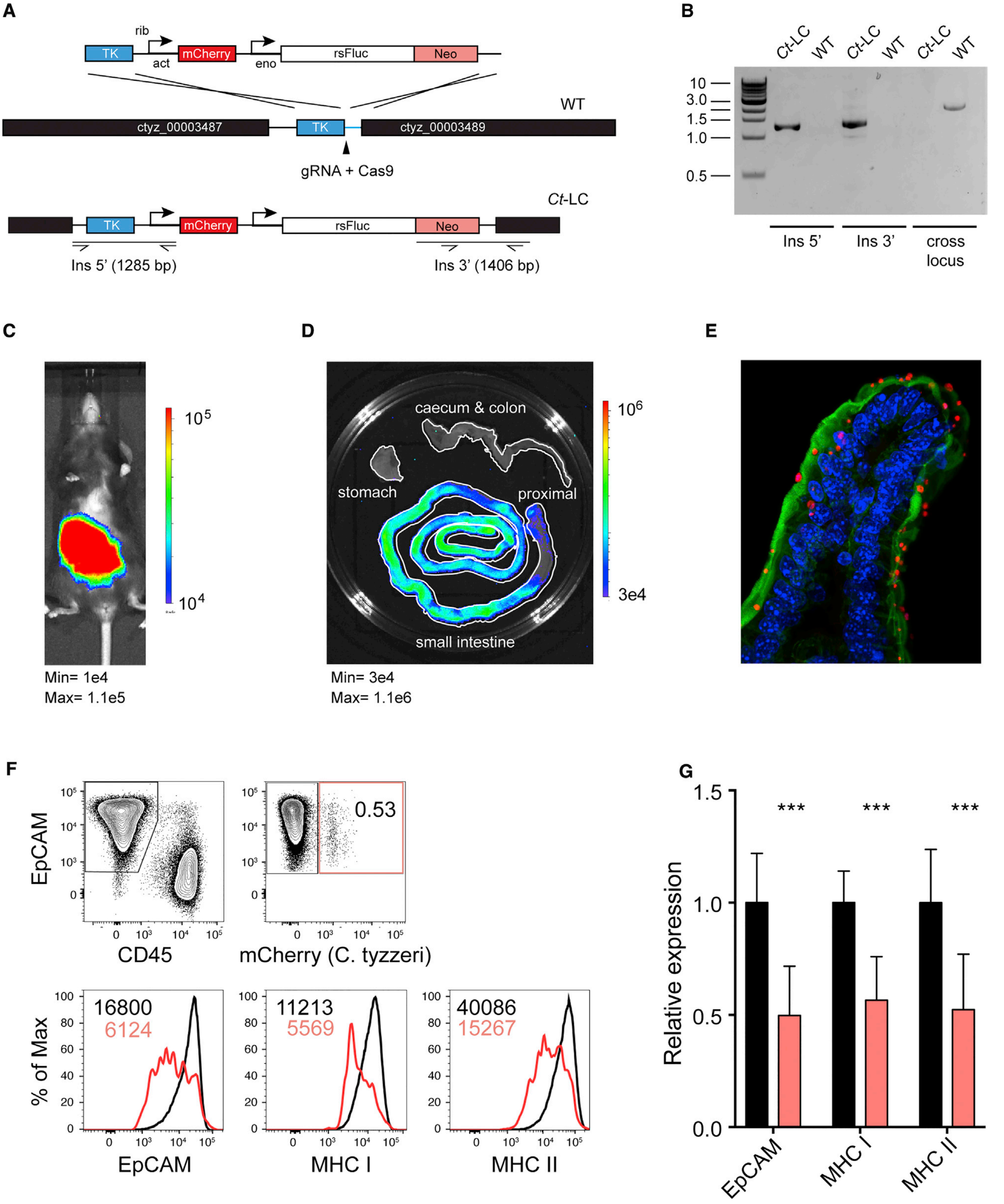

Figure 4. Genetic Manipulation of *Cryptosporidium tyzzeri*

(A) Map of the thymidine kinase (TK) locus targeted in *C. tyzzeri* by insertion of a construct that includes the selection marker Neomycin phosphotransferase (Neo), redshifted firefly luciferase (rsFLuc), and the fluorescent protein reporter mCherry. The solid black arrow indicates the position of the Cas9 induced double-strand break.

(legend continued on next page)

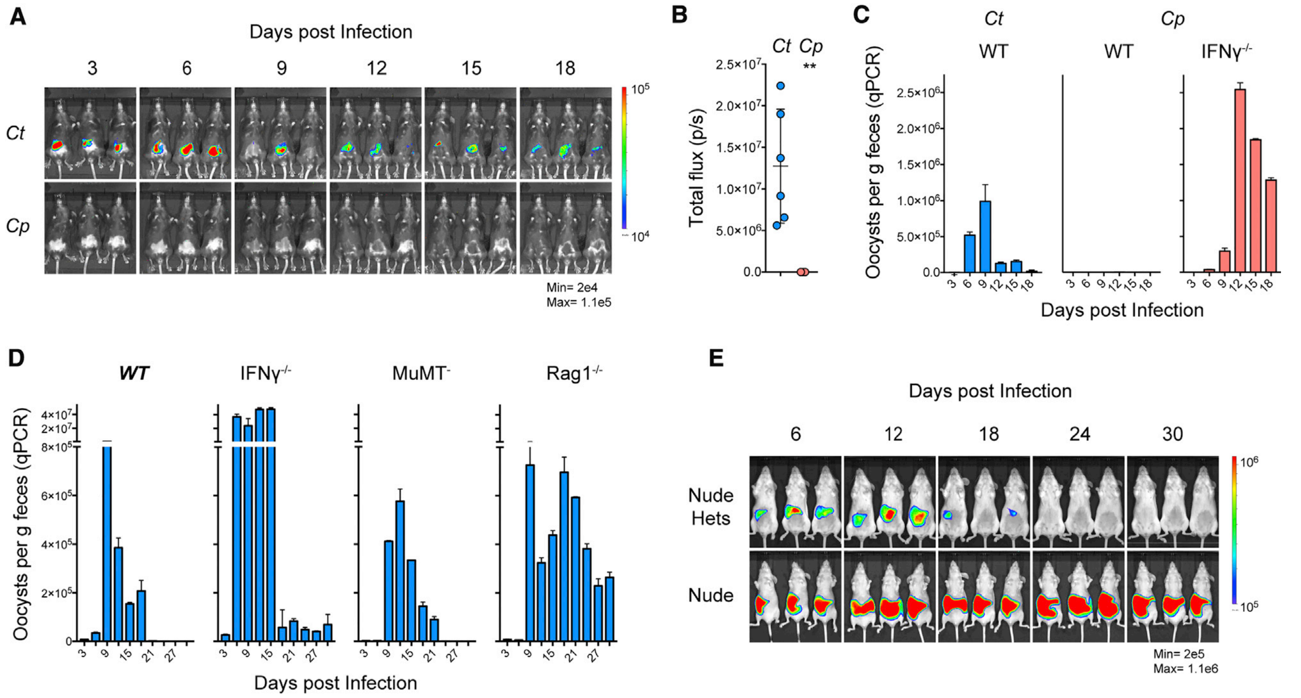

Figure 5. Interferon γ and T Cells Govern Susceptibility and Resolution of Murine Cryptosporidiosis

(A–C) Immunocompetent C57BL/6 mice infected with 10,000 oocysts of *C. tyzzeri* (*Ct*) or *C. parvum* (*Cp*). Infection was monitored by whole animal imaging following parasite luciferase (A, quantified over entire infection in B) or by qPCR detecting parasite shed in the feces (C). Note that Cp produces robust infection in Ifn$\gamma^{-/-}$. For A and B, n = 9 mice total over 2 experiments, images shown are representative, and data represent mean ± SD with significance determined using a two-tailed t test (*p < 0.05). For C, n = 12 mice total, 4 per group, with qPCR performed on pooled fecal material with two technical replicates.

(D) C57BL/6 wild-type mice (WT) and strains lacking IFNγ (Ifn$\gamma^{-/-}$), mature B cells (μMt^{-}), or mature B and T cells (Rag1$^{-/-}$) were infected with 10,000 *C. tyzzeri* oocysts and infection was monitored by qPCR from collected feces. n = 16 mice total, 4 per group, with qPCR performed on pooled fecal material with two technical replicates, and data represent mean ± SD.

(E) Homozygous (Nude) and heterozygous (Nude Hets) Foxn1^{nu} mice were infected with 10,000 transgenic *C. tyzzeri* oocysts and monitored for one month. n = 6 mice total; quantification of luminescence and fecal parasite shedding by qPCR in Figure S3. Mice lacking T cells (Rag1$^{-/-}$ and Nude) fail to clear the infection.

A vaccine to prevent or limit the severity of cryptosporidiosis could significantly reduce child mortality and impact stunting and its many detrimental consequences (Khalil et al., 2018). Previous studies in mice have used *Salmonella* vectors to deliver *Cryptosporidium* antigens with limited efficacy (Bartelt et al., 2016) and perhaps the use of irradiated oocysts in calves has proven the most effective strategy (Jenkins et al., 2004). Indeed, parasites attenuated through irradiation, natural mutation, or genetic engineering show promising activity for related parasites including *Toxoplasma* and *Plasmodium* (Fox and Bzik, 2002; Mueller et al., 2005). Therefore, to assess whether *C. tyzzeri* would provide a system to establish a live-attenuated vaccine, wild-type *C. tyzzeri* oocysts were subjected to increasing doses of gamma irradiation and used to infect C57BL/6 mice. Irradiation at 200gray decreased total parasite shedding in mice to 11% of non-irradiated controls, and at doses of 300gray and above parasite shedding was not detected (Figure S5A). Mice that received parasites attenuated with irradiation at 300gray demonstrated significant protection when rechallenged with transgenic Ct-LC parasites (Figures 6E, 6F, and S5B–S5F). Single or multiple exposures to this attenuated parasite vaccine led to protection equal to that observed in mice that experienced a live infection. To begin to define the requirements for vaccine induced immunity we conducted vaccination studies in T cell or IFNγ-deficient mouse strains lacking the key players we identified for resolution of the infection. In contrast to wild-type C57BL/6, *Rag1*$^{-/-}$ and *Ifn*$\gamma^{-/-}$ mice showed no protection from challenge following multiple vaccine exposures (Figures 6G and 6H). These results underscore the central role of IFNγ and T cells in resistance to *Cryptosporidium* (Figure 6I) and they

(B) PCR analysis using genomic DNA from wild-type (WT) and transgenic (Ct-LC) parasites. Note that insertion of the multi-gene cassette into the locus precludes amplification across the locus because of the excessive size of the amplicon. Therefore, amplification is only apparent in WT.

(C–E) The resulting transgenic strain, Ct-LC, can be used to locate and measure parasite burden in (C) the whole animal, (D) the intestinal tract, and (E) in infected tissue (immunofluorescence of small intestine with red-mCherry, green-actin, blue-nuclei).

(F and G) Small intestines of infected mice were disaggregated into single cell suspension and analyzed by flow cytometry. EpCAM and CD45 distinguish enterocytes and leukocytes (F). Infected enterocytes are identified by mCherry and comparison of uninfected (black) and infected (red) cells harvested from the same animal show downregulation of EpCAM and MHC I and II (G). Data are mean ± SD and significance is determined using a Student's t test comparing infected and uninfected epithelial cells (***p < 0.001).

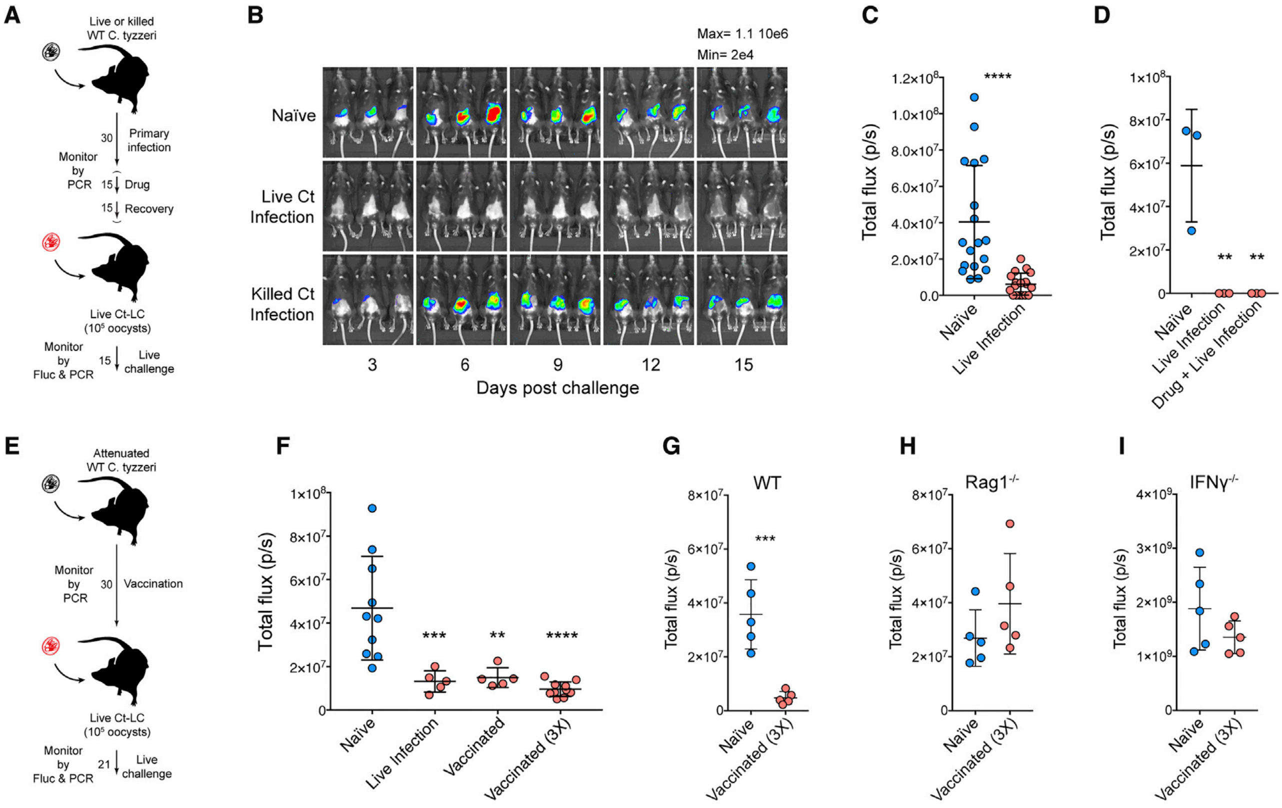

Figure 6. Infection Results in Protective Immunity That Can Be Induced with an Attenuated Vaccine

(A) Schematic outline of challenge experiments for B, C, and D.

(B) C57BL/6 mice were subjected to primary infection as indicated, followed by a challenge with 100,000 oocysts of transgenic *C. tyzzeri*. n = 9 total mice, 3 per group.

(C) Summary of parasite burden (total flux over the two-week imaging period) comparing naive and previously infected mice. n = 36 total mice, 18 per group, over 5 independent experiments. Data represent mean ± SD with significance determined using a Students t test (****$p < 0.00005$).

(D) Parasite burden of challenge with and without intervening paromomycin treatment. n = 9 total mice, 3 per group, and data represent mean ± SD with significance determined using one-way ANOVA with Dunnett multiple comparison test (**$p < 0.005$). Individual parasite burden by imaging and fecal parasite shedding by qPCR in Figure S4.

(E) Schematic outline of vaccination experiments for F–I.

(F) Mice were infected or vaccinated as indicated and then challenged with 100,000 transgenic *C. tyzzeri* oocysts. Total parasite burden from whole animal imaging is shown. Individual parasite burden by imaging and fecal parasite shedding by qPCR in Figure S5. Note that live infection and vaccination results in comparable protection. n = 30 mice, with each dot representing the total parasite burden for a single mouse. Data are mean ± SD and significance is determined using one-way ANOVA with Dunnett multiple comparisons test (**$p < 0.005$, ***$p < 0.0005$, and ****$p < 0.00005$).

(G–I) Mice were vaccinated with oocysts attenuated by 300 gray of gamma irradiation then challenged with 1000,000 transgenic *C. tyzzeri* oocysts. Total parasite burden from whole animal imaging is shown. Note the large difference in parasite tissue burden of mice deficient IFNγ (I) compared to both WT C57BL/6 mice (G) and mice that lack mature B and T cells (H). n = 10 mice for each strain, with each dot representing the total parasite burden for a single mouse. Data are mean ± SD and significance is determined using Student's t test comparing naive and vaccinated mice within each strain (***$p < 0.0005$).

show that this natural model provides an experimental system to rigorously define the host and parasite factors required for a vaccine to generate the pathogen-specific T cells necessary to protect from cryptosporidiosis.

DISCUSSION

Cryptosporidium is an important pathogen with a global public health impact, but the numerous technical difficulties to study *Cryptosporidium* have limited progress (Striepen, 2013). However, recent advances in genetic manipulation (Vinayak et al., 2015), cryo-preservation of viable parasites (Jaskiewicz et al., 2018), and production of infectious oocyst within intestinal organoid cultures (Heo et al., 2018) have transformed the ability to manipulate *Cryptosporidium*. In the infective oocyst stage, the parasite is sheltered by a chemically and environmentally resilient wall that renders water chlorination ineffective in protecting drinking and recreation water, in fact we routinely use bleach in the laboratory when purifying oocysts to kill bacteria and viruses with no detriment to the parasite. Outbreaks, even in countries with advanced water treatment efforts, are common and can occur at massive scale (>460,000 cases in the 1993 Milwaukee outbreak; Mac Kenzie et al., 1994). In the United States, for example, despite the efforts of the Centers of Disease Control and others the incidence of cryptosporidiosis has been steady if not rising in recent years (Painter et al., 2015). Studies in India

found that providing bottled water alone did little to curb cryptosporidiosis in children in a high endemicity setting (Sarkar et al., 2013). While improved water sanitation is of obvious benefit to pediatric health, additional tools are needed to protect children from cryptosporidiosis and others have highlighted the potential impact of a vaccine (Khalil et al., 2018). The model of cryptosporidiosis reported here replicates many features of human infection in a system where parasite and host are genetically tractable and provides the opportunity to develop a rational approach to test different vaccination strategies.

Cryptosporidiosis has epidemiological and clinical overlap with rotavirus infection, as both are common infections of young children (Kotloff et al., 2013). Several rotavirus vaccines are now available, including formulations optimized for populations outside of the United States and Europe. As an example, India has developed and now produces its own vaccine (Bhandari et al., 2014). These vaccines are safe and effective, and their use is recommended by the WHO (2013). A vaccine for cryptosporidiosis could have a similar life-saving impact for children in low and medium income countries. Rotavirus vaccines do not afford complete protection (Bar-Zeev et al., 2015; Bhandari et al., 2014), but nonetheless carry significant benefit. Similarly, natural infection with rotavirus does not result in sterile immunity yet provides protection from subsequent severe disease (Velázquez et al., 1996). This is remarkably similar to cryptosporidiosis; children can experience multiple infections and human volunteers can be infected in a secondary challenge, however, older children and adults with prior exposure show enhanced protection from disease (Chappell et al., 1999; Korpe et al., 2018; Kotloff et al., 2013). Infection with *C. tyzzeri* accurately matches the level of immunity observed in children, not sterile and yet significantly protective, providing a realistic test for vaccine candidates in a natural host-parasite relationship.

C. tyzzeri produces self-limiting infections restricted to the small intestine, and while mice do not develop watery diarrhea in response to this (or most other) infections, the associated pathology closely resembles what is seen in humans. Villus blunting, along with loss of the apical glycocalyx and effacement of microvilli can be readily detected in response to infection. There is also significant increase in the turnover of enterocytes manifesting as crypt hyperplasia. This could reflect an anaplerotic response simply replenishing cells lost because of infection. Alternatively, this could also be part of an orchestrated physiological response to limit the parasite through an accelerated villus elevator leading to enhanced shedding. While such physiological mechanisms are now a well-established as response to intestinal nematodes (Cliffe et al., 2005), examples for a role in the control of intracellular pathogens (Rauch et al., 2017) are still rare.

In the intestine the immune system faces important challenges that it has to balance. Microbes are extremely abundant as part of the commensal flora and they and their products must not trigger inflammation to avoid autoimmunity and allergy. At the same time, the intestine is a major entry point for infections and the immune system has to mount a vigorous response in an overall tolerogenic environment. This challenge may be reflected in the lack of ready sterile immunity to intracellular intestinal pathogens, but ultimately this is overcome. This natural mouse model of an infection that is truly limited to the intestinal epithelium offers a unique opportunity to mechanistically understand how immunity can overcome this challenge and may allow us to discovery ways to manipulate the process to better prevent disease.

The mouse model of cryptosporidiosis presented here allows for the study of immunity and pathogenesis in mice with mature and fully functional immune systems. Mouse strains with specific immune deficiencies can now be infected and compared against appropriate wild-type control. Two key players have already emerged from this early study: T cells and IFNγ. T cells are required to clear a primary infection as well as for the protective effect of the vaccine. IFNγ was previously implicated in innate control of *C. parvum* in mice and humans (Griffiths et al., 1998; White et al., 2000) and we similarly observe an important role early in *C. tyzzeri* infection. We further note that mice lacking IFNγ fail to fully clear *C. tyzzeri* infection and show no protection when vaccinated. IFNγ thus plays a central and complex role in both innate and adaptive immunity to *Cryptosporidium*, but the mechanism by which IFNγ acts to restrict *Cryptosporidium* within an infected enterocyte is unknown (Pollok et al., 2001). Another poorly understood aspect of *Cryptosporidium* immunity is which types of parasite antigen would be appropriate targets for protective immunity. Cryptosporidium occupies a peculiar, intracellular yet extracellular, position at the very periphery of the epithelium, and parasites are shed into the intestinal lumen and it is unclear what cellular processes are involved in antigen sampling and presentation required for T cell priming. The enterocyte itself could have an important role in this process, as they do express MHC class I and II, but their ability to prime CD4 and CD8 T cells in the context of an enteric pathogen has not been tested. In addition, there are specialized sub-sets of dendritic cells in the gut that have been shown to be able to sample microbial antigens from the lumen (Niess et al., 2005; Rescigno et al., 2001). It is now possible to assess their contribution to role in the development of *Cryptosporidium* specific T cell responses during infection or vaccination.

Recently, there has been a vigorous and concerted effort to develop new and more effective *Cryptosporidium* treatments (Baragaña et al., 2019; Buckner et al., 2019; Lee et al., 2019; Love et al., 2017; Manjunatha et al., 2017). One barrier to drug development is the high cost of *in vivo* drug testing; immunocompromised mice, gnotobiotic piglets, and calves are all suitable yet expensive models to test drug efficacy. *Cryptosporidium tyzzeri* infects a wide range of inbred and outbred mice (data not shown) and thus will lower the bar to measuring efficacy of compounds. Furthermore, malnutrition, a common complication in cryptosporidiosis infection, treatment, and recovery, can be effectively modeled using the natural mouse model (Costa et al., 2011). We note that while there is a significant change in fecal consistency during infection, mice do not develop watery diarrhea during *C. tyzzeri* infection. Because of this limitation, calves remain the model of cryptosporidiosis to evaluate the impact of diarrhea on pharmacokinetics.

C. tyzzeri is, phylogenetically, very closely related to the parasites that cause human infection, *C. hominis* and *C. parvum*. In fact, our *de novo* assembly for *C. tyzzeri* indicated that over 99% of its genome aligns with the genome sequences of *C. hominis* and *parvum* with >75% nucleotide identity. This high degree of conservation suggests that much of the parasite

biology and metabolism are shared, which will facilitate *in vivo* analysis when investigating the function of specific genes and pathways. Despite the high level of identity and synteny, there are differences and they are most apparent in polymorphic regions that encode families of secretory proteins of still largely unknown function (Abrahamsen et al., 2004; Templeton et al., 2004; Xu et al., 2004). These differences may hold answers to the question of what determines host specificity. Mice are not a natural host for *C. parvum*, yet can be infected when immunosuppressed, most pronounced when IFNγ is removed. This suggests that, at least in part, the difference between *C. tyzzeri* and *C. parvum* may be explained by the ability of the parasite to appropriately interact with a host's innate immune response. Proteins or protein families that distinguish parasite species thus may be strong candidates for factors that act to modulate or overcome immunity and their diversification is likely driven by a coevolution of host-parasite interaction that favors specialization. The ability to genetically engineer both *C. parvum* and *C. tyzzeri* offers an opportunity to experimentally dissect the molecular basis of their interaction with their murine host.

STAR★METHODS

Detailed methods are provided in the online version of this paper and include the following:

- KEY RESOURCES TABLE
- CONTACT FOR REAGENT AND RESOURCE SHARING
- EXPERIMENTAL MODEL AND SUBJECT DETAILS
  - Mouse Models of Infection
  - Parasite Strains
- METHOD DETAILS
  - Isolation of Parasites
  - 18S Phylogenetic Analyses
  - Genome Sequencing
  - Genome Assembly and Annotation
  - SNP Analyses and SNP/Synteny Map Generation
  - Generation of Transgenic Parasites
  - Measuring Parasite Shedding by Fecal qPCR
  - Measuring Parasite Burden in Tissue by In Vivo Imaging
  - Measuring Parasite Burden in Tissue by qPCR
  - Flow Cytometry
  - Histology of Intestinal Tissue
  - Immunofluorescence of Intestinal Tissue
  - Gamma Irradiation of Parasites
- QUANTIFICATION AND STATISTICAL ANALYSIS
- DATE AND SOFTWARE AVAILABILITY

SUPPLEMENTAL INFORMATION

Supplemental Information can be found online at https://doi.org/10.1016/j.chom.2019.05.006.

ACKNOWLEDGMENTS

We thank Dr. Lorraine Fuller for help and advice on oocyst collection and isolation and Dr. Cameron Koch for access to and help with gamma irradiation. This work was funded in part by grants from the National Institutes of Health (R01AI112427) and the Bill & Melinda Gates Foundation to B.S. (OPP1161001) and to J.C.K. (OPP1151701). A.S. was supported by a fellowship (F32AI124518) and a pathway to independence award (K99AI137442), and J.A.G. was supported by a fellowship (T32A1055400) from the National Institutes of Health.

AUTHOR CONTRIBUTIONS

J.S. and A.S. collected field samples, isolated oocysts, and conducted initial passage and typing. A.S. developed transgenic model, sequenced genome, and conducted mouse studies into susceptibility, resolution, immunity, and vaccination with support from G.T.H., C.F.B., and E.M.K. R.B. and J.C.K. assembled and annotated the genome sequence and performed comparative analyses, J.B.E. conducted histopathological analysis and J.A.G. and C.A.H. flow cytometry. A.S., J.S., and B.S. conceived of the study and A.S. and B.S. wrote the manuscript with contributions from all authors.

DECLARATION OF INTERESTS

The authors declare no competing interests.

Received: March 15, 2019
Revised: April 30, 2019
Accepted: May 15, 2019
Published: June 20, 2019

SUPPORTING CITATIONS

The following references appear in the Supplemental Information: Emms and Kelly (1992); Guindon et al. (2010); Stamatakis (2014).

REFERENCES

WHO (2013). Rotavirus vaccines WHO position paper: January 2013-recommendations. Vaccine *31*, 6170–6171.

Abrahamsen, M.S., Templeton, T.J., Enomoto, S., Abrahante, J.E., Zhu, G., Lancto, C.A., Deng, M., Liu, C., Widmer, G., Tzipori, S., et al. (2004). Complete genome sequence of the apicomplexan, Cryptosporidium parvum. Science *304*, 441–445.

Baragaña, B., Forte, B., Choi, R., Nakazawa Hewitt, S., Bueren-Calabuig, J.A., Pisco, J.P., Peet, C., Dranow, D.M., Robinson, D.A., Jansen, C., et al. (2019). Lysyl-tRNA synthetase as a drug target in malaria and cryptosporidiosis. Proc. Natl. Acad. Sci. USA *116*, 7015–7020.

Bartelt, L.A., Bolick, D.T., Kolling, G.L., Roche, J.K., Zaenker, E.I., Lara, A.M., Noronha, F.J., Cowardin, C.A., Moore, J.H., Turner, J.R., et al. (2016). Cryptosporidium priming is more effective than vaccine for protection against cryptosporidiosis in a murine protein malnutrition model. PLoS Negl. Trop. Dis. *10*.

Bar-Zeev, N., Kapanda, L., Tate, J.E., Jere, K.C., Iturriza-Gomara, M., Nakagomi, O., Mwansambo, C., Costello, A., Parashar, U.D., Heyderman, R.S., et al. (2015). Effectiveness of a monovalent rotavirus vaccine in infants in Malawi after programmatic roll-out: an observational and case-control study. Lancet Infect. Dis. *15*, 422–428.

Belkaid, Y., Piccirillo, C.A., Mendez, S., Shevach, E.M., and Sacks, D.L. (2002). CD4+CD25+ regulatory T cells control Leishmania major persistence and immunity. Nature *420*, 502–507.

Bhandari, N., Rongsen-Chandola, T., Bavdekar, A., John, J., Antony, K., Taneja, S., Goyal, N., Kawade, A., Kang, G., Rathore, S.S., et al. (2014). Efficacy of a monovalent human-bovine (116E) rotavirus vaccine in Indian infants: a randomised, double-blind, placebo-controlled trial. Lancet *383*, 2136–2143.

Boetzer, M., and Pirovano, W. (2012). Toward almost closed genomes with GapFiller. Genome Biol. *13*, R56.

Boetzer, M., and Pirovano, W. (2014). SSPACE-LongRead: scaffolding bacterial draft genomes using long read sequence information. BMC Bioinformatics *15*, 211.

Bolger, A.M., Lohse, M., and Usadel, B. (2014). Trimmomatic: a flexible trimmer for Illumina sequence data. Bioinformatics *30*, 2114–2120.

Borad, A., and Ward, H. (2010). Human immune responses in cryptosporidiosis. Future Microbiol. *5*, 507–519.

Buckner, F.S., Ranade, R.M., Gillespie, J.R., Shibata, S., Hulverson, M.A., Zhang, Z., Huang, W., Choi, R., Verlinde, C.L.M.J., Hol, W.G.J., et al. (2019). Optimization of methionyl tRNA-synthetase inhibitors for treatment of cryptosporidium infection. Antimicrob. Agents Chemother. *63*.

Cantarel, B.L., Korf, I., Robb, S.M., Parra, G., Ross, E., Moore, B., Holt, C., Sánchez Alvarado, A., and Yandell, M. (2008). MAKER: an easy-to-use annotation pipeline designed for emerging model organism genomes. Genome Res. *18*, 188–196.

Chappell, C.L., Okhuysen, P.C., Sterling, C.R., Wang, C., Jakubowski, W., and Dupont, H.L. (1999). Infectivity of Cryptosporidium parvum in healthy adults with pre-existing anti-C. parvum serum immunoglobulin G. Am. J. Trop. Med. Hyg. *60*, 157–164.

Checkley, W., Epstein, L.D., Gilman, R.H., Black, R.E., Cabrera, L., and Sterling, C.R. (1998). Effects of Cryptosporidium parvum infection in Peruvian children: growth faltering and subsequent catch-up growth. Am. J. Epidemiol. *148*, 497–506.

Checkley, W., White, A.C., Jr., Jaganath, D., Arrowood, M.J., Chalmers, R.M., Chen, X.M., Fayer, R., Griffiths, J.K., Guerrant, R.L., Hedstrom, L., et al. (2015). A review of the global burden, novel diagnostics, therapeutics, and vaccine targets for cryptosporidium. Lancet Infect. Dis. *15*, 85–94.

Choudhry, N., Korbel, D.S., Edwards, L.A., Bajaj-Elliott, M., and McDonald, V. (2009). Dysregulation of interferon-gamma-mediated signalling pathway in intestinal epithelial cells by Cryptosporidium parvum infection. Cell. Microbiol. *11*, 1354–1364.

Cliffe, L.J., Humphreys, N.E., Lane, T.E., Potten, C.S., Booth, C., and Grencis, R.K. (2005). Accelerated intestinal epithelial cell turnover: a new mechanism of parasite expulsion. Science *308*, 1463–1465.

Costa, L.B., JohnBull, E.A., Reeves, J.T., Sevilleja, J.E., Freire, R.S., Hoffman, P.S., Lima, A.A.M., Oriá, R.B., Roche, J.K., Guerrant, R.L., et al. (2011). Cryptosporidium-malnutrition interactions: mucosal disruption, cytokines, and TLR signaling in a weaned murine model. J. Parasitol. *97*, 1113–1120.

Edgar, R.C. (2004). MUSCLE: multiple sequence alignment with high accuracy and high throughput. Nucleic Acids Res. *32*, 1792–1797.

Emms, D.M., and Kelly, S. (2015). OrthoFinder: solving fundamental biases in whole genome comparisons dramatically improves orthogroup inference accuracy. Genome Biol. *16*, 157.

Flanigan, T., Whalen, C., Turner, J., Soave, R., Toerner, J., Havlir, D., and Kotler, D. (1992). Cryptosporidium infection and CD4 counts. Ann. Intern. Med. *116*, 840–842.

Fox, B.A., and Bzik, D.J. (2002). De novo pyrimidine biosynthesis is required for virulence of Toxoplasma gondii. Nature *415*, 926–929.

Griffiths, J.K., Theodos, C., Paris, M., and Tzipori, S. (1998). The gamma interferon gene knockout mouse: a highly sensitive model for evaluation of therapeutic agents against Cryptosporidium parvum. J. Clin. Microbiol. *36*, 2503–2508.

Gu, Z., Gu, L., Eils, R., Schlesner, M., and Brors, B. (2014). Circlize Implements and enhances circular visualization in R. Bioinformatics *30*, 2811–2812.

Guindon, S., Dufayard, J.F., Lefort, V., Anisimova, M., Hordijk, W., and Gascuel, O. (2010). New algorithms and methods to estimate maximum-likelihood phylogenies: assessing the performance of PhyML 3.0. Syst. Biol. *59*, 307–321.

Heiges, M., Wang, H., Robinson, E., Aurrecoechea, C., Gao, X., Kaluskar, N., Rhodes, P., Wang, S., He, C.Z., Su, Y., et al. (2006). CryptoDB: a Cryptosporidium bioinformatics resource update. Nucleic Acids Res. *34*, D419–D422.

Heo, I., Dutta, D., Schaefer, D.A., Iakobachvili, N., Artegiani, B., Sachs, N., Boonekamp, K.E., Bowden, G., Hendrickx, A.P.A., Willems, R.J.L., et al. (2018). Modelling Cryptosporidium infection in human small intestinal and lung organoids. Nat. Microbiol. *3*, 814–823.

Huston, C.D., Spangenberg, T., Burrows, J., Willis, P., Wells, T.N.C., and van Voorhis, W. (2015). A proposed target product profile and developmental cascade for new cryptosporidiosis treatments. PLoS Negl. Trop. Dis. *9*, e0003987.

Jaskiewicz, J.J., Sandlin, R.D., Swei, A.A., Widmer, G., Toner, M., and Tzipori, S. (2018). Cryopreservation of infectious Cryptosporidium parvum oocysts. Nat. Commun. *9*, 2883.

Jenkins, M., Higgins, J., Kniel, K., Trout, J., and Fayer, R. (2004). Protection of calves against cryptosporiosis by oral inoculation with gamma-irradiated Cryptosporidium parvum oocysts. J. Parasitol. *90*, 1178–1180.

Johnson, A.D., Handsaker, R.E., Pulit, S.L., Nizzari, M.M., O'Donnell, C.J., and de Bakker, P.I.W. (2008). SNAP: a web-based tool for identification and annotation of proxy SNPs using HapMap. Bioinformatics *24*, 2938–2939.

Jokipii, L., and Jokipii, A.M. (1986). Timing of symptoms and oocyst excretion in human cryptosporidiosis. N. Engl. J. Med. *315*, 1643–1647.

Jothikumar, N., da Silva, A.J., Moura, I., Qvarnstrom, Y., and Hill, V.R. (2008). Detection and differentiation of Cryptosporidium hominis and Cryptosporidium parvum by dual TaqMan assays. J. Med. Microbiol. *57*, 1099–1105.

Khalil, I.A., Troeger, C., Rao, P.C., Blacker, B.F., Brown, A., Brewer, T.G., Colombara, D.V., De Hostos, E.L., Engmann, C., Guerrant, R.L., et al. (2018). Morbidity, mortality, and long-term consequences associated with diarrhoea from Cryptosporidium infection in children younger than 5 years: a meta-analyses study. Lancet Glob. Health *6*, e758–e768.

Korpe, P.S., Gilchrist, C., Burkey, C., Taniuchi, M., Ahmed, E., Madan, V., Castillo, R., Ahmed, S., Arju, T., Alam, M., et al. (2018). Case-control study of cryptosporidium transmission in Bangladeshi households. Clin. Infect. Dis.

Kotloff, K.L., Nataro, J.P., Blackwelder, W.C., Nasrin, D., Farag, T.H., Panchalingam, S., Wu, Y., Sow, S.O., Sur, D., Breiman, R.F., et al. (2013). Burden and aetiology of diarrhoeal disease in infants and young children in developing countries (the Global Enteric Multicenter Study, GEMS): a prospective, case-control study. Lancet *382*, 209–222.

Krzywinski, M., Schein, J., Birol, I., Connors, J., Gascoyne, R., Horsman, D., Jones, S.J., and Marra, M.A. (2009). Circos: an information aesthetic for comparative genomics. Genome Res. *19*, 1639–1645.

Kumar, S., Stecher, G., and Tamura, K. (2016). MEGA7: molecular evolutionary genetics analysis 7.0 version for bigger datasets. Mol. Biol. Evol. *33*, 1870–1874.

Kváč, M., McEvoy, J., Loudová, M., Stenger, B., Sak, B., Květoňová, D., Ditrich, O., Rašková, V., Moriarty, E., Rost, M., et al. (2013). Coevolution of Cryptosporidium tyzzeri and the house mouse (Mus musculus). Int. J. Parasitol. *43*, 805–817.

Lee, S., Ginese, M., Girouard, D., Beamer, G., Huston, C.D., Osbourn, D., Griggs, D.W., and Tzipori, S. (2019). Piperazine-Derivative MMV665917: an effective drug in the diarrheic piglet model of Cryptosporidium hominis. J. Infect. Dis.

Li, H., and Durbin, R. (2009). Fast and accurate short read alignment with Burrows-Wheeler transform. Bioinformatics *25*, 1754–1760.

Li, H., Handsaker, B., Wysoker, A., Fennell, T., Ruan, J., Homer, N., Marth, G., Abecasis, G., and Durbin, R.; Genome Project Data Processing (2009). The sequence alignment/map format and SAMtools. Bioinformatics *25*, 2078–2079.

Lomsadze, A., Ter-Hovhannisyan, V., Chernoff, Y.O., and Borodovsky, M. (2005). Gene identification in novel eukaryotic genomes by self-training algorithm. Nucleic Acids Res. *33*, 6494–6506.

Love, M.S., Beasley, F.C., Jumani, R.S., Wright, T.M., Chatterjee, A.K., Huston, C.D., Schultz, P.G., and McNamara, C.W. (2017). A high-throughput phenotypic screen identifies clofazimine as a potential treatment for cryptosporidiosis. PLoS Negl. Trop. Dis. *11*, e0005373.

Lumadue, J.A., Manabe, Y.C., Moore, R.D., Belitsos, P.C., Sears, C.L., and Clark, D.P. (1998). A clinicopathologic analysis of AIDS-related cryptosporidiosis. AIDS *12*, 2459–2466.

Mac Kenzie, W.R., Hoxie, N.J., Proctor, M.E., Gradus, M.S., Blair, K.A., Peterson, D.E., Kazmierczak, J.J., Addiss, D.G., Fox, K.R., and Rose, J.B. (1994). A massive outbreak in Milwaukee of cryptosporidium infection transmitted through the public water supply. N. Engl. J. Med. *331*, 161–167.

Manabe, Y.C., Clark, D.P., Moore, R.D., Lumadue, J.A., Dahlman, H.R., Belitsos, P.C., Chaisson, R.E., and Sears, C.L. (1998). Cryptosporidiosis in patients with AIDS: correlates of disease and survival. Clin. Infect. Dis. *27*, 536–542.

Manjunatha, U.H., Vinayak, S., Zambriski, J.A., Chao, A.T., Sy, T., Noble, C.G., Bonamy, G.M.C., Kondreddi, R.R., Zou, B., Gedeck, P., et al. (2017). A Cryptosporidium PI(4) K inhibitor is a drug candidate for cryptosporidiosis. Nature *546*, 376–380.

Martina, B.E.E., Haagmans, B.L., Kuiken, T., Fouchier, R.A.M., Rimmelzwaan, G.F., Van Amerongen, G., Peiris, J.S.M., Lim, W., and Osterhaus, A.D.M.E. (2003). Virology: SARS virus infection of cats and ferrets. Nature *425*, 915.

McKenna, A., Hanna, M., Banks, E., Sivachenko, A., Cibulskis, K., Kernytsky, A., Garimella, K., Altshuler, D., Gabriel, S., Daly, M., et al. (2010). The genome analysis toolkit: a MapReduce framework for analyzing next-generation DNA sequencing data. Genome Res. *20*, 1297–1303.

Mead, J.R. (2010). Challenges and prospects for a Cryptosporidium vaccine. Future Microbiol. *5*, 335–337.

Mead, J.R. (2014). Prospects for immunotherapy and vaccines against Cryptosporidium. Hum. Vaccin. Immunother. *10*, 1505–1513.

Mead, J.R., Arrowood, M.J., Sidwell, R.W., and Healey, M.C. (1991). Chronic Cryptosporidium parvum infections in congenitally immunodeficient SCID and nude mice. J. Infect. Dis. *163*, 1297–1304.

Mølbak, K., Andersen, M., Aaby, P., Højlyng, N., Jakobsen, M., Sodemann, M., and da Silva, A.P. (1997). Cryptosporidium infection in infancy as a cause of malnutrition: a community study from Guinea-Bissau, west Africa. Am. J. Clin. Nutr. *65*, 149–152.

Mondal, D., Haque, R., Sack, R.B., Kirkpatrick, B.D., and Petri, W.A., Jr. (2009). Attribution of malnutrition to cause-specific diarrheal illness: evidence from a prospective study of preschool children in Mirpur, Dhaka, Bangladesh. Am. J. Trop. Med. Hyg. *80*, 824–826.

Mueller, A.K., Labaied, M., Kappe, S.H.I., and Matuschewski, K. (2005). Genetically modified Plasmodium parasites as a protective experimental malaria vaccine. Nature *433*, 164–167.

Niess, J.H., Brand, S., Gu, X., Landsman, L., Jung, S., McCormick, B.A., Vyas, J.M., Boes, M., Ploegh, H.L., and Fox, J.G. (2005). CX3CR1-mediated dendritic cell access to the intestinal lumen and bacterial clearance. Science *307*, 254–258.

Okhuysen, P.C., Chappell, C.L., Sterling, C.R., Jakubowski, W., and DuPont, H.L. (1998). Susceptibility and serologic response of healthy adults to reinfection with Cryptosporidium parvum. Infect. Immun. *66*, 441–443.

Otto, T.D., Sanders, M., Berriman, M., and Newbold, C. (2010). Iterative correction of reference nucleotides (iCORN) using second generation sequencing technology. Bioinformatics *26*, 1704–1707.

Painter, J.E., Hlavsa, M.C., Collier, S.A., Xiao, L., and Yoder, J.S.; Centers for Disease, C., and Prevention (2015). Cryptosporidiosis surveillance – United States, 2011–2012. MMWR Suppl. *64*, 1–14.

Pawlowic, M.C., Vinayak, S., Sateriale, A., Brooks, C.F., and Striepen, B. (2017). Generating and maintaining transgenic Cryptosporidium parvum parasites. Curr. Protoc. Microbiol. *46*, 20B 22 21–20B 22 32.

Petry, F., Robinson, H.A., and McDonald, V. (1995). Murine infection model for maintenance and amplification of Cryptosporidium parvum oocysts. J. Clin. Microbiol. *33*, 1922–1924.

Platts-Mills, J.A., Babji, S., Bodhidatta, L., Gratz, J., Haque, R., Havt, A., McCormick, B.J.J., McGrath, M., Olortegui, M.P., Samie, A., et al. (2015). Pathogen-specific burdens of community diarrhoea in developing countries: a multisite birth cohort study (MAL-ED). Lancet Glob. Health *3*, E564–E575.

Ploss, A., Evans, M.J., Gaysinskaya, V.A., Panis, M., You, H., de Jong, Y.P., and Rice, C.M. (2009). Human occludin is a hepatitis C virus entry factor required for infection of mouse cells. Nature *457*, 882–886.

Pollok, R.C.G., Farthing, M.J.G., Bajaj-Elliott, M., Sanderson, I.R., and McDonald, V. (2001). Interferon gamma induces enterocyte resistance against infection by the intracellular pathogen Cryptosporidium parvum. Gastroenterology *120*, 99–107.

Quinlan, A.R., and Hall, I.M. (2010). BEDTools: a flexible suite of utilities for comparing genomic features. Bioinformatics *26*, 841–842.

Rauch, I., Deets, K.A., Ji, D.X., von Moltke, J., Tenthorey, J.L., Lee, A.Y., Philip, N.H., Ayres, J.S., Brodsky, I.E., Gronert, K., et al. (2017). NAIP-NLRC4 Inflammasomes coordinate intestinal epithelial cell expulsion with eicosanoid and IL-18 release via activation of caspase-1 and -8. Immunity *46*, 649–659.

Ren, X., Zhao, J., Zhang, L., Ning, C., Jian, F., Wang, R., Lv, C., Wang, Q., Arrowood, M.J., and Xiao, L. (2012). Cryptosporidium tyzzeri n. sp. (Apicomplexa: Cryptosporidiidae) in domestic mice (Mus musculus). Exp. Parasitol. *130*, 274–281.

Rescigno, M., Urbano, M., Valzasina, B., Francolini, M., Rotta, G., Bonasio, R., Granucci, F., Kraehenbuhl, J.P., and Ricciardi-Castagnoli, P. (2001). Dendritic cells express tight junction proteins and penetrate gut epithelial monolayers to sample bacteria. Nat. Immunol. *2*, 361–367.

Sarkar, R., Ajjampur, S.S.R., Prabakaran, A.D., Geetha, J.C., Sowmyanarayanan, T.V., Kane, A., Duara, J., Muliyil, J., Balraj, V., Naumova, E.N., et al. (2013). Cryptosporidiosis among children in an endemic semiurban community in southern India: does a protected drinking water source decrease infection? Clin. Infect. Dis. *57*, 398–406.

Schmidt, W., Wahnschaffe, U., Schäfer, M., Zippel, T., Arvand, M., Meyerhans, A., Riecken, E.O., and Ullrich, R. (2001). Rapid increase of mucosal CD4 T cells followed by clearance of intestinal cryptosporidiosis in an AIDS patient receiving highly active antiretroviral therapy. Gastroenterology *120*, 984–987.

Stamatakis, A. (2014). RAxML version 8: a tool for phylogenetic analysis and post-analysis of large phylogenies. Bioinformatics *30*, 1312–1313.

Stanke, M., and Morgenstern, B. (2005). Augustus: a web server for gene prediction in eukaryotes that allows user-defined constraints. Nucleic Acids Res. *33*, W465–467.

Striepen, B. (2013). Parasitic infections: time to tackle cryptosporidiosis. Nature *503*, 189–191.

Templeton, T.J., Iyer, L.M., Anantharaman, V., Enomoto, S., Abrahante, J.E., Subramanian, G.M., Hoffman, S.L., Abrahamsen, M.S., and Aravind, L. (2004). Comparative analysis of Apicomplexa and genomic diversity in eukaryotes. Genome Res. *14*, 1686–1695.

Tzipori, S. (1998). Cryptosporidiosis: laboratory investigations and chemotherapy. Adv. Parasitol. *40*, 187–221.

Velázquez, F.R., Matson, D.O., Calva, J.J., Guerrero, M.L., Morrow, A.L., Carter-Campbell, S., Glass, R.I., Estes, M.K., Pickering, L.K., and Ruiz-Palacios, G.M. (1996). Rotavirus infection in infants as protection against subsequent infections. N. Engl. J. Med. *335*, 1022–1028.

Vinayak, S., Pawlowic, M.C., Sateriale, A., Brooks, C.F., Studstill, C.J., Bar-Peled, Y., Cipriano, M.J., and Striepen, B. (2015). Genetic modification of the diarrhoeal pathogen Cryptosporidium parvum. Nature *523*, 477–480.

White, A.C., Robinson, P., Okhuysen, P.C., Lewis, D.E., Shahab, I., Lahoti, S., DuPont, H.L., and Chappell, C.L. (2000). Interferon-gamma expression in jejunal biopsies in experimental human cryptosporidiosis correlates with prior sensitization and control of oocyst excretion. J. Infect. Dis. *181*, 701–709.

Widmer, G., Akiyoshi, D., Buckholt, M.A., Feng, X., Rich, S.M., Deary, K.M., Bowman, C.A., Xu, P., Wang, Y., Wang, X., et al. (2000). Animal propagation and genomic survey of a genotype 1 isolate of Cryptosporidium parvum. Mol. Biochem. Parasitol. *108*, 187–197.

Xu, P., Widmer, G., Wang, Y., Ozaki, L.S., Alves, J.M., Serrano, M.G., Puiu, D., Manque, P., Akiyoshi, D., Mackey, A.J., et al. (2004). The genome of Cryptosporidium hominis. Nature *431*, 1107–1112.

STAR★METHODS

KEY RESOURCES TABLE

REAGENT or RESOURCE	SOURCE	IDENTIFIER
Antibodies		
anti-I-A/I-E	BioLegend	Clone M5/114.15.2; RRID: AB_2565976
anti-EpCAM	BioLegend	Clone G8.8; RRID: AB_1134105
anti-H-2Db	BioLegend	Clone KH95; RRID: AB_313510
anti-CD45.2	eBioscience	Clone 104; RRID: AB_2534956
anti-H-2Kb	BD Biosciences	Clone AF6-88.5; RRID: AB_394927
Alexa Fluor 488 Phalloidin	Thermofisher	Cat# A12379; RRID: AB_2315147
Rat monoclonal anti-mCherry	Thermofisher	Clone 16D7; RRID: AB_2536611
Goat-anti-Rat polyclonal Alexa Fluor 594	Thermofisher	Cat# A-11007; RRID: AB_10561522
Critical Commercial Assays		
Quick-DNA Fecal/Soil Microbe Kit	Zymo Research	Cat# D6010
anti-*Cryptosporidium* magnetic beads	Thermofisher	Cat# 73011
DNeasy Blood & Tissue Kit	Qiagen	Cat# 69504
Deposited Data		
Raw data from WGS of *Cryptosporidium tyzzeri*	NCBI	SRR5683558
Assembled and annotated genome	EuPathDB	UGA55
Experimental Models: Organisms/Strains		
Cryptosporidium tyzzeri	This paper	UGA55 strain
Cryptosporidium parvum	Bunchgrass Farms, ID	IOWA strain
C57BL/6 mice	Jackson Laboratory	Strain 000664
Ifng$^{-/-}$ mice	Jackson Laboratory	Strain 002287
Rag1$^{-/-}$ mice	Jackson Laboratory	Strain 002216
μMt^{-} mice	Jackson Laboratory	Strain 002288
athymic mice	Jackson Laboratory	Strain 002019
Oligonucleotides		
Primers for cloning, see Table S2	This paper	N/A
18 s probe and primer for parasite quantification, see Table S2	Jothikumar et al., 2008	N/A
Recombinant DNA		
Cas9 expression vector with *Cryptosporidium tyzzeri* thymidine kinase locus guide	This paper and Vinayak et al., 2015	N/A
Cryptosporidium mCherry/RE9 expression cassette vector	This paper	N/A
Software and Algorithms		
MEGA software package	Kumar et al., 2016	https://www.megasoftware.net/
Genome Analysis Toolkit v3.8.0	McKenna et al., 2010	https://software.broadinstitute.org/gatk/
Picard tools v2.16.0	https://broadinstitute.github.io/picard/	https://broadinstitute.github.io/picard/
Burrows-Wheeler Aligner	Li and Durbin, 2009	http://bio-bwa.sourceforge.net/
Bedtools v2.26	Quinlan and Hall, 2010	https://github.com/arq5x/bedtools2
Samtools v1.6	Li et al., 2009	http://samtools.sourceforge.net/
R with circos package	Gu et al., 2014	https://www.r-project.org/
PRISM	GraphPad	https://www.graphpad.com/scientific-software/prism/
FlowJo v10 software	TreeStar	https://www.flowjo.com/

(*Continued on next page*)

Continued		
REAGENT or RESOURCE	SOURCE	IDENTIFIER
Other		
137-Cs irradiator	J.L Shepard	Mark1-30
LSRFortessa	BD Biosciences	N/A
IVIS	Caliper Life Sciences	Lumina II
ViiA 7 Real-Time System	Thermofisher	N/A

CONTACT FOR REAGENT AND RESOURCE SHARING

For access to reagents or parasite strains used in this study please contact the Lead Contact, Boris Striepen (striepen@upenn.edu).

EXPERIMENTAL MODEL AND SUBJECT DETAILS

Mouse Models of Infection

C57BL/6 (stock no:000664), Ifng$^{-/-}$ (stock no:002287), Rag1$^{-/-}$ (stock no:002216), μMt^{-} (stock no:002288), and athymic mice (stock no:002019) were purchased from Jackson Laboratory. Unless otherwise noted, mice used in this study were females ranging from 4 – 8 weeks, however, we note that we did not measure a difference in infection burden between male and female mice in both C57BL/6 and Ifng$^{-/-}$ mice. All mice were gender and age matched within individual experiments. All protocols for animal care were approved by the Institutional Animal Care and Use Committee of the University of Georgia (protocol A2016 01-028-Y1-A4) and the Institutional Animal Care and Use Committee of the University of Pennsylvania (protocol #806292).

Parasite Strains

Cryptosporidium tyzzeri wild-type and transgenic strains are propagated within infected Ifng$^{-/-}$ mice (stock no:002287). Oocysts are then purified from fecal collections using sucrose flotation followed by a cesium chloride gradient (see Method Details). *Cryptosporidium parvum* oocysts used in this study are Iowa strain, purchased from Bunchgrass Farms (Dreary, ID).

METHOD DETAILS

Isolation of Parasites

Mouse fecal pellets were collected and stored in water overnight. Collected pellets were then assayed by qPCR for the presence of the *Cryptosporidium* using a 18S probe and primer set (see Table S2) from fecal isolated with Quick-DNA Fecal/Soil Microbe Kit (Zymo Research, Irvine, CA, USA). If positive for the presence of *Cryptosporidium*, sample DNA was subjected to further genotyping (gp60, SSU, and COWP1) to determine the species. During this time fecal pellets were stored in 2.5% potassium dichromate at 4°C. To inoculate mice, fecal samples were first washed 3× with cold water to remove potassium dichromate, then filtered through a 200um strainer. Strained fecal material was treated with a 1:4 dilution of household bleach for 5min on ice, washed 3× with cold tap water, and used to gavage C57BL/6 mice housed in quarantine. Parasite shedding was monitored by qPCR, and infected mice underwent a full screen by a veterinary pathologist to test for concurrent infections (see Table S1). This procedure was repeated twice to transfer the infection to successive cages, with an additional purification step utilizing anti-*Cryptosporidium* magnetic beads (Thermofisher, Waltham, MA). Again, mice underwent a full screen by a veterinary pathologist following each infection.

18S Phylogenetic Analyses

18S rRNA from UGA55 was amplified using specific primers (see Table S2) and phylogenetic tree was created with the MEGA software package (Kumar et al., 2016). Alignment was performed using MUSCLE (Edgar, 2004) under default settings, and phylogenetic reconstruction was performed using Maximum Likelihood, Neighbor-Joining, and Maximum Parsimony to ensure consensus. Maximum Likelihood is displayed in the manuscript using 500 bootstrap replications. Sequences used for alignment are as follows: *Cryptosporidium fragilis* GenBank: EU162753.1, *Cryptosporidium andersoni* GenBank: AY954885.1, *Cryptosporidium muris* GenBank: L19069.1, *Cryptosporidium felis* GenBank: AF108862.1, *Cryptosporidium canis* GenBank: AF112576.1, *Cryptosporidium meleagridis* GenBank: AF112574.1, *Cryptosporidium wrairi* GenBank: AF115378.1, *Cryptosporidium baileyi* GenBank: L19068.1, *Cryptosporidium suis* GenBank: AF115377.1, *Cryptosporidium fayari* GenBank: AF108860.1, *Cryptosporidium hominis* CryptoDB: Chro.rrn016, and *Cryptosporidium parvum* CryptoDB: cgd7_7.

Genome Sequencing

10^7 *C. tyzzeri* oocysts were incubated at 37°C for 1 hr in 0.8% sodium taurocholate in PBS to induce excystation. DNA was then purified using a Qiagen DNeasy Blood & Tissue Kit (Qiagen, Hilden, Germany), and a DNA library was prepared with a Nextera XT Prep

Kit (Illumina, San Diego, CA, USA). Reads were generated using 150-bp pared-end sequencing on a MiSeq platform (Illumina, San Diego, CA, USA) at the Georgia Genomics and Bioinformatics Core.

Genome Assembly and Annotation

All reads obtained by the Illumina Miseq sequencing platform had their adapters and low-quality sequences removed using (Bolger et al., 2014). The threshold used was a minimum read length of 100 bases and a phred-scale quality score > 30. The genome was assembled *de novo* with Velvet assembler v1.2.10 using a k-mer value of 87 and a minimum contig length of 800 bases. Scaffolding was performed using two lines of evidence, firstly using the paired-end information of 300 insert size followed by a guided scaffolding using the close related *C. parvum* genome PacBio long reads to order these resulted scaffolds from the previous step using SSPACE-long reads v.1.1 (Boetzer and Pirovano, 2014). The final scaffolds were submitted to GapFiller v.1.10 (Boetzer and Pirovano, 2012) with 10 interactions to extend end fill the gaps using the Illumina sequence, and each base was corrected by using ICORN v.0.97 (Otto et al., 2010).

The genome was annotated using three different gene prediction tools: (i) an *ab initio* prediction using GeneMark-ES (Lomsadze et al., 2005); (ii) evidence-trained predictions by SNAP/Maker (Cantarel et al., 2008; Johnson et al., 2008) and (iii) Augustus (Stanke and Morgenstern, 2005). The training-based step for these predictions was made by using evidence from publicly available data from several *Cryptosporidium* species and a manual curation of all genes in the context of existing molecular evidence (Baptista et al., unpublished data).

SNP Analyses and SNP/Synteny Map Generation

Cryptosporidium Illumina reads (Sequence Read Archive accession numbers: SRR5683558 *C.tyzzeri* UGA55; SRR1557959 *C.hominis* 30976; SRR6147945 *C. parvum* UKP6; and SRR001350 *C. muris* RN66) were mapped against the *C. tyzzeri* UGA55 genome sequence generated in this project using BWA v. 0.7.15 (Li and Durbin, 2009) with the mem mode. The resulting alignment was then processed to remove PCR duplicates (which can introduce bias in variant calling) and also increases the quality and reliability of the analysis using Picard tools v2.16.0 (http://broadinstitute.github.io/picard/). Variants were called using the Genome Analysis Toolkit v3.8.0 (GATK) Haplotypecaller (McKenna et al., 2010) with a ploidy of one. Variants with low mapping quality (<25), allele frequency lower than 70%, or less than 10x coverage were discarded. The variant call files (vcf) were then processed using samtools v1.6 (Li et al., 2009) and bedtools v.2.26 (Quinlan and Hall, 2010). SNPs were then grouped into 1 kb windows and plotted in R using the circlize package (Gu et al., 2014). For synteny analysis, genome sequences were aligned using blastn with an e-value of $1e^{-20}$, and then the alignments were plotted using the Rcircos package for R (Krzywinski et al., 2009).

Generation of Transgenic Parasites

10^7 *C. tyzzeri* oocysts were first incubated at 37°C for 1 hr in 0.8% sodium taurocholate to induce excystation. Excysted sporozoites were then transfected using an Amaxa 4D electroporater (Lonza, Basel, Switzerland) with parasites suspended in SF buffer and using the program EH100. 50 μg of each plasmid were used for transfection, one encoding Cas9/gRNA, and another encoding a selection cassette surrounded by 1 kb of homologous DNA for insertion into the genome (Pawlowic et al., 2017; Vinayak et al., 2015). Transfected parasites were surgically implanted (Vinayak et al., 2015) into the small intestine of anesthetized $Ifng^{-/-}$ mice. Paromomycin, was given to the mice in their drinking water (16 mg/mL) ad libitum to select for parasites rendered resistant through expression of neomycin phosphotransferase. Parasite shedding was monitored by fecal qPCR and feces were collected daily during peak shedding.

To purify transgenic parasites from collected feces we used sucrose flotation followed by a cesium chloride gradient (Pawlowic et al., 2017). Collected mouse feces was homogenized in tap water using a LabGen 125 homogenizer (Cole-Parmer, Vernon Hills, IL, USA) and filtered through a 250-μm mesh filter. This resulting filtrate was mixed 1:1 with a saturated sucrose solution (specific gravity 1.33) and centrifuged at 1,000 g for 5 min. Supernatant was collected, resuspended in 0.85% saline solution and overlaid onto CsCl solution (specific gravity 1.15), and centrifuged at 16,000 g for 3 min. Purified oocysts were collected from the saline-CsCl interface and resuspended in cold PBS.

Measuring Parasite Shedding by Fecal qPCR

Parasite DNA was isolated from 100 mg of collected fecal material using a Quick-DNA Fecal/Soil Microbe Kit (Zymo Research, Irvine, CA, USA). Parasite shedding was measured using a *Cryptosporidium* specific probe and primer set (Jothikumar et al., 2008) (see Table S2). SsoAdvanced Universal Probes Supermix (BioRad, Hercules, CA, USA) was used with a ViiA 7 Real-Time System (Thermofisher, Waltham, MA, USA) to perform qPCR. To quantify parasites samples were run with a standard curve of DNA from fecal samples spiked with a known concentration of oocysts.

Measuring Parasite Burden in Tissue by In Vivo Imaging

Mice were subcutaneously injected with D-luciferin (Gold Biotechnology, St Louis, MO, USA) at 125 mg/kg, then anesthetized in an induction chamber using 2.5% isoflurane. After five minutes mice were moved into an IVIS Lumina II instrument (Caliper Life Sciences, Waltham, MA, USA) and luminescence was measured using 5 min exposure, medium binning, and 1/16 F-stop.

Measuring Parasite Burden in Tissue by qPCR

Mice were killed according to our approved animal use protocol and sections were dissected from the small intestine and colon, 1 cm in length, and thoroughly washed in PBS. From the stomach and the caecum, 1 cm x 1 cm square sections were cut. DNA was purified from each section using a Qiagen DNeasy Blood & Tissue Kit (Qiagen, Hilden, Germany) and parasite burden was measured using a *Cryptosporidium* specific probe and primer set (see Table S2). SsoAdvanced Universal Probes Supermix (BioRad, Hercules, CA, USA) was used with a ViiA 7 Real-Time System (Thermofisher, Waltham, MA, USA) to perform qPCR.

Flow Cytometry

Single-cell suspensions were prepared from intestinal sections by shaking diced tissue at 37°C for 25 minutes in Hank's Balanced Salt Solution with 5 mM EDTA and 1 mM DTT. Cell pellets were then passed through 70 μm and 40 μm filters. Cells were surface stained using the following fluorochrome-conjugated Abs: anti-I-A/I-E (M5/114.15.2), anti-EpCAM (G8.8), and anti-H-2Db (KH95) from BioLegend, anti-CD45.2 (104) from eBioscience, and anti-H-2Kb (AF6-88.5) from BD Biosciences. Data were collected on a LSRFortessa (BD Biosciences) and were analyzed with FlowJo v10 software (TreeStar).

Histology of Intestinal Tissue

Mice were killed and small intestine was dissected. This tissue was flushed with 10% neutral buffered formalin (Sigma, St Louis, MO, USA), then 'swiss-rolled' and fixed overnight. Fixed samples were paraffin-embedded, sectioned, and stained with haematoxylin and eosin. Slides were evaluated by a board-certified veterinary pathologist in a blinded fashion for quantitative measurements of number of parasites, villus/crypt architectural features and inflammatory infiltrates, and semi-quantitative scores for villus epithelium lesions (see Figure S6). Intestinal segments with highest number of parasites comprising distal jejunum to proximal ileum, which were consistent for all mice in the study, were selected for detailed histologic evaluation. To avoid inconsistent intra-segmental measurements due to differing orientations of villi and crypts within the section, five consecutive and complete villus-crypt structures, meaning villus and crypt lumens within the same plane of section, were selected for scoring. *C. tyzzeri* organisms within the apical regions of villus epithelium within each of the five villi were totaled. Measurements obtained for villus height, villus width taken at the mid-point of the villus height, crypt depth and crypt width taken at the mid-depth of the crypt were averaged, and villus:crypt height ratios were calculated. Numbers of villus and crypt goblet cells, crypt mitotic figures and lamina propria inflammatory infiltrates (neutrophils, eosinophils, plasma cells) for 5 villus/crypt structures were tallied and averaged. Gastro-Intestinal Lymphoid Tissue (GALT) foci and non-follicular lymphocyte aggregates for each section of distal jejunum/ileum segments were also enumerated. Semi-quantitative scores for the following parameters were generated as follows: villus epithelial separation/sloughing (0 = none; 1 = cell separation from villus tip of 1-2 out of 5 villi evaluated; 2 = cell separation from villus tip of 3-5 out of 5 villi evaluated; 3 = denuded villus tips; 4 = denuded villus tips with fibrin and/or inflammatory exudate); villus epithelial dysplasia (0 = none; cells well-aligned in a monolayer; 1 = cytoplasmic basophilia with high nuclear:cytoplamic ratio; 2 = mild, multifocal epithelial disorganization; 3 = regional moderate epithelial malalignment, 4 = marked regional malalignment with cell piling); villus epithelial attenuation (0 = none, epithelium are uniformly tall and columnar-shaped; 1 = multifocal cuboidal-shaped epithelium; 2 = regional-multiregional cuboidal shaped-epithelium; 3 = flattened villus epithelium); and degree of crypt branching (0 = none; 1 = 1-2 branches observed for 1–2 out of 5 crypts evaluated; 2 = 1-2 branches observed for 3-5 out of 5 crypts evaluated; 3 = greater than 2 branches within 1 or more crypts out of 5 crypts evaluated.

Immunofluorescence of Intestinal Tissue

Mice were killed and the distal 1/3 of the small intestine was dissected. This tissue was flushed with 10% neutral buffered formalin (Sigma, St Louis, MO, USA), then 'swiss-rolled' and fixed overnight. Samples were then placed 30% sucrose in phosphate buffered saline (PBS) for cryoprotection, and mounted with OCT compound (Tissue-Tek, Sakura Finetek, Japan). Sections were permeabilized and blocked with 10% BSA in PBS with 0.1% triton-x (Sigma, St Louis, MO, USA) for 1 hr, then incubated with primary and secondary antibody in 1% BSA in PBS with 0.1% triton-x. Primary label used: 1:50 Alexa Fluor 647 Phalloidin (Thermofisher, Waltham, MA), 1:1000 rat monoclonal anti-mCherry, 1-50 rabbit polyclonal anti-CD3 (Abcam, Cambridge, UK). Secondary antibodies used: Goat-anti-Rat polyclonal Alexa Fluor 594 (Thermofisher, Waltham, MA) and Goat-anti-Rabbit polyclonal Alexa Fluor 488 (Thermofisher, Waltham, MA). Nuclei of parasite and host were stained using Hoeschst 33342 (Thermofisher, Waltham, MA).

Gamma Irradiation of Parasites

Oocysts were exposed to gamma irradiation using a J.L Shepard Mark 1-30 self-shielded irradiator with a ^{137}Cs source and were kept cold throughout within a chilled tube rack.

QUANTIFICATION AND STATISTICAL ANALYSIS

GraphPad PRISM was used for all statistical analyses. When measuring the difference between two populations, we used a standard T-test. For data sets with 3 or more experimental groups we used a one-way ANOVA with Dunnett's multiple comparison's test. No

statistical tests were used to predetermine sample size and no animals were excluded from results. Replicates were as follows: Figure 3A, N = 7 from two separate experiments; Figure 3D–3H, N = 20 with 5 mice per experimental group; Figures 5A and 5B, N = 9 mice from two separate experiments; Figures 4F and 4G, for EpCAM measurements n = 16 mice from 6 experiments, for MHCI measurements n = 8 mice from 3 experiments, for MHCII measurements n = 12 mice from 5 experiments; Figure 5C, N = 12 with 4 mice per experimental group; Figure 5D, N = 16 with 4 mice per experimental group; Figure 5E, N = 6 with 3 mice per experimental group; Figure 6A, N = 9 with 3 mice per experimental group; Figure 6C, N = 36 with 18 mice per experimental group over 5 separate experiments; Figure 6D, N = 9 with 3 mice per experimental group; Figure 6F, N = 30 mice over two separate experiments; Figure 6G, N = 10 with 5 mice per experimental group; Figure 6H, N = 10 with 5 mice per experimental group; Figure 6I, N = 10 with 5 mice per experimental group.

DATE AND SOFTWARE AVAILABILITY

Raw sequencing data is available through NCBI (SRR5683558) and a fully assembled and annotated genome of *Cryptosporidium tyzzeri* is available through the Eukaryotic Pathogen Database (https://eupathdb.org/eupathdb/). Please refer to the Key Resources Table for links to the software used for genome assembly, annotation, and analysis.

Cell Host & Microbe, Volume *26*

Supplemental Information

A Genetically Tractable, Natural Mouse Model of Cryptosporidiosis Offers Insights into Host Protective Immunity

Adam Sateriale, Jan Šlapeta, Rodrigo Baptista, Julie B. Engiles, Jodi A. Gullicksrud, Gillian T. Herbert, Carrie F. Brooks, Emily M. Kugler, Jessica C. Kissinger, Christopher A. Hunter, and Boris Striepen

Table S1: Comprehensive diagnostic panel for quarantined mice, related to Figure 1

Laboratory animal parasitology panel	Laboratory animal pathogen PCR diagnostics	Laboratory animal mite PCR	Laboratory animal pinworm PCR
Direct exam	Mouse parvovirus	*Myobia musculi*	*Aspicularis tetraptera*
Fecal float	*Mycoplasma pulomonis*	*Myocoptes musculinis*	*Syphacia muris*
Anal tape	Lymphocytic choriomeningitis		
Ectoparasite exam	Mouse Adenovirus		
	Ectromelia virus		
	Polyoma virus		
	Minute virus of mice		
	Mouse hepatitis virus		
	Clostridium piliforme		
	Theiler's encephalomyelitis virus		
	Mouse rotavirus		
	Sendai virus		
	Pneumonia virus of mice		
	Reovirus		

Table S2: Sequences of primers and probes used in this study, related to Star Methods

Purpose	Primer/probe name	Sequence (5′ to 3′)
qPCR detection of Cryptosporidium	18S-JVA-F1	ATGACGGGTAACGGGGAAT
	18S-JVA-R1	CCAATTACAAAACCAAAAAGTCC
	18S-JVA-probe	[FAM]CGCGCCTGCTGCCTTCCTTAGATG[BHQ1]
PCR diagnostics of genetically manipulated locus	TK5F1	TTCCTCTTCCTTATTATACCCACCTAA
	TK3R1	CAGCTTCTTCTCCACCTGATAATATAGTATCTGTACC
	NeoF1	CGTATGCCCGACGGTGAAGATCTTG
	ActinR1	CTATTATTACTGTTTATACCAACTCATTCTGAAG
PCR diagnostics of collected mouse fecal pellets	BtkF1	AATGGGCTAGCGTAGTGCAG
	BtkR1	AGGGGACGTACACTCAGCTTT
	Zfy2F1	CATTAAGACAGAAAAGAC
	Zfy2R1	GTGAGGAAATTTCTTCCT
Guide RNA sequence for Cas9	TKendF	GTTGGAAGAATATAATCTCTGAGG
	TKendR	AAACCCTCAGAGATTATATTCTTC

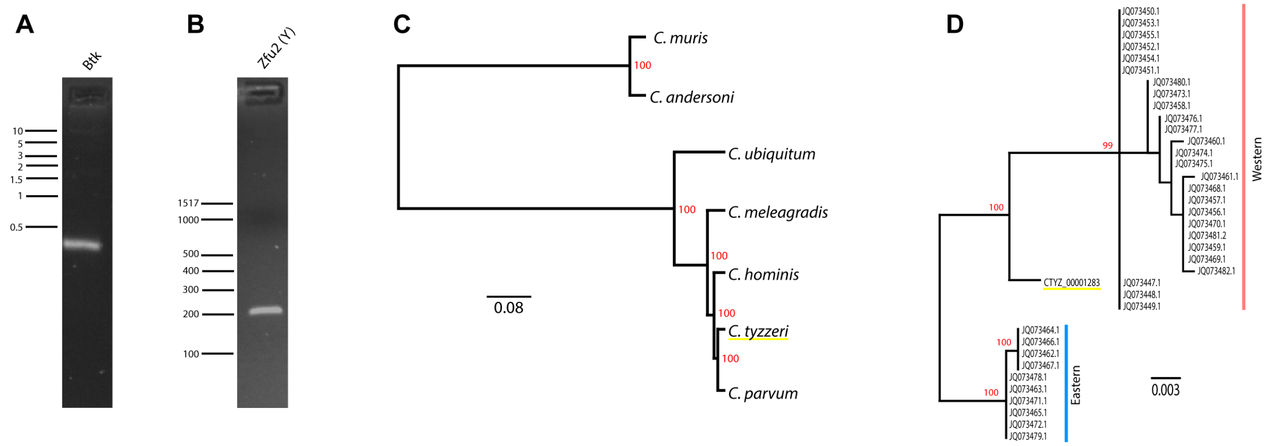

Figure S1: PCR and phylogenetic analysis, related to Figure 1 (A-B) PCR of DNA from field collected fecal pellets identifies *Mus musculus domestius* as natural *C. tyzzeri* host. **(A)** Gel of PCR using primers specific for a 342 bp segment of the Bruton agammaglobulinemia tyrosine kinase gene (Btk). This gene is present on the X chromosome of *Mus musculus domesticus*, but absent in *M. m. musculus*. **(B)** PCR using primers for the Zfu2 marker, which amplifies a 202 bp segment of both zinc finger protein 1 (Zfy1), and zinc finger protein 2 (Zfy2) on the Y chromosome. The genome of *M. m. musculus* has an 18 bp deletion in the last exon of Zfy2, producing two bands (202 and 184), whereas *M. m. domesticus* produces a single band (202). **(C-D)** Phylogenetic analysis of *Cryptosporidium tyzzeri.* **(C)** Whole genome phylogenetic tree constructed using 2764 single copy orthologous genes from *Cryptosporidium* species for which a full genome sequence data is available (available for download at CryptoDB.org). Single gene orthologs were identified using OrthoFinder (Emms and Kelly, 2015) and the tree was constructed using PhyML (Guindon et al., 2010) with JTT+1 settings and 1000 bootstraps for topology statistics. Genomes used for this analysis were: *Cryptosporidium muris* RN66, *Cryptosporidium andersoni* isolate 30847, *Cryptosporidium ubiquitum* isolate 39726, *Cryptosporidium meleagradis* strain UKMEL1, *Cryptosporidium hominis* 30976, *Cryptosporidium tyzzeri* UGA55, and *Cryptosporidium parvum* Iowa II. **(D)** Phylogenetic tree comparing gp60 sequences from *Cryptosporidium tyzzeri* UGA55 (CTYZ_00001283) and those PCR amplified from mice on the Eastern and Western side of the *M. m. musculus–M. m. domesticus* hybrid zone spanning from the Czech Republic to Germany see Kvac et al. (Kvac et al., 2013) for sequences and further detail. The alignments were constructed with MAFFT (Katoh and Standley, 2013), the phylogenetic tree was constructed with RaXML (Stamatakis, 2014) with 100 bootstrap replicates. Only branches with significant support are labeled. We note that using the current data set we direct lineage to either the Eastern or Western European *C. tyzzeri* cannot be assigned.

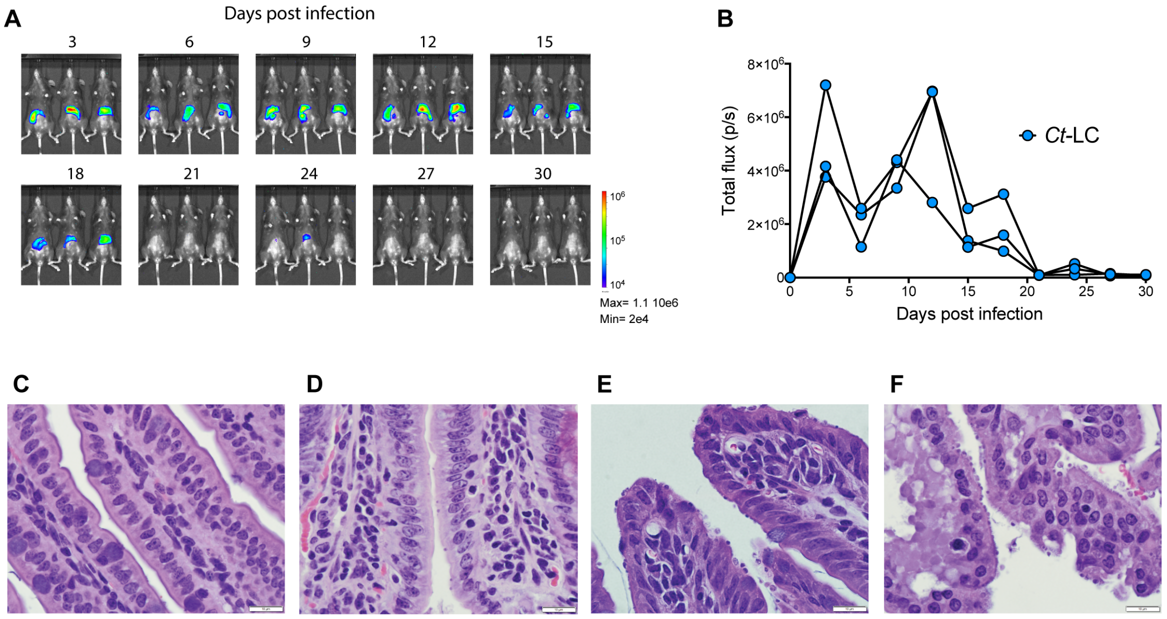

Figure S2: Whole animal imaging with Ct-LC parasites and histology of infected ileum sections, related to Figure 5 (A-B) Luminescence imaging of C57BL/6 mice infected with *Ct*-LC parasites. **(A)** Whole animal imaging of C57BL/6 mice infected with 10,000 *Ct*-LC oocysts as shown in Figure 3. **(B)** Parasite tissue burden represented as total flux (p/s) measurements. Each line represents a single mouse. **(C-F)** Histology of Ifn$\gamma^{-/-}$ and C57BL/6 mice. Hematoxylin and eosin stained sections of ilial tissue taken from mice on the tenth day of *Cryptosporidium* infection. Mice were 8 weeks old at the time of infection and were inoculated with 50,000 oocysts by gavage. **(C)** C57BL/6 mice infected with *Cryptosporidium parvum*, **(D)** C57BL/6 mice infected with *Cryptosporidium tyzzeri*, **(E)** Ifn$\gamma^{-/-}$ mice infected with *Cryptosporidium parvum*, and **(F)** Ifn$\gamma^{-/-}$ mice infected with *Cryptosporidium tyzzeri*.

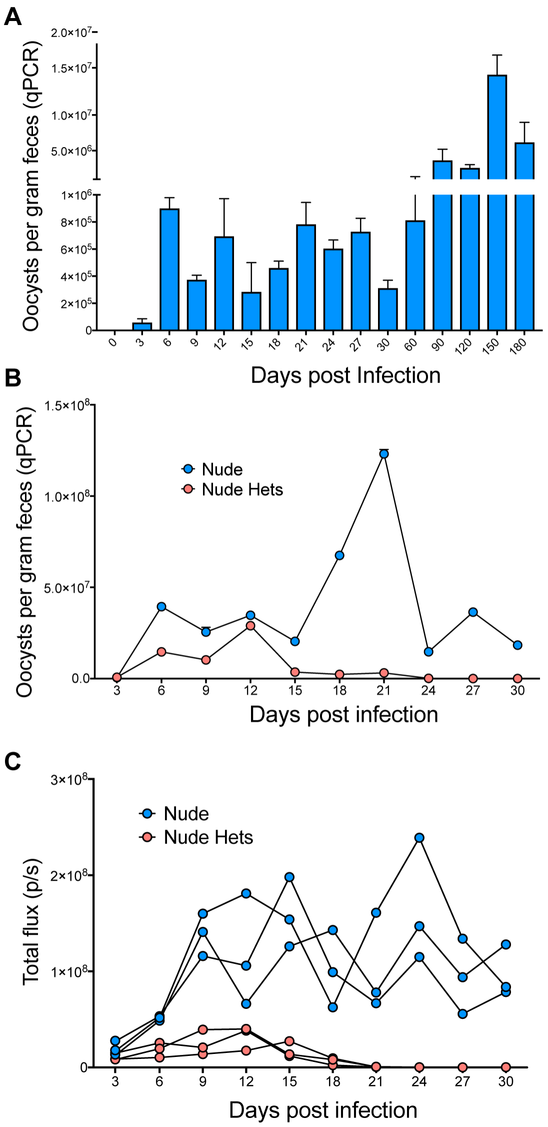

Figure S3: Infection in Rag$^{-/-}$ and athymic mice, related to Figure 5 (A) Extended time course of parasite burden in Rag$^{-/-}$ mice. Eight week old Rag1$^{-/-}$ mice infected with 50,000 *C. tyzzeri* oocysts each. Parasite shedding measured by qPCR from DNA isolated from pooled fecal samples. Two technical replicates and data shown is mean $\pm$ s.d., and *n* = 4 mice. **(B-C)** Fecal shedding and luminescence imaging of athymic mice. Foxn1^{nu} mice were infected with 10,000 transgenic *Ct*-LC oocysts. **(B)** Parasite shedding measured by qPCR from pooled fecal collections. Two technical replicates and data shown is mean $\pm$ s.d.. **(C)** Parasite tissue burden measured by whole animal imaging for individual mice. *n* = 6 mice total, 3 mice per group.

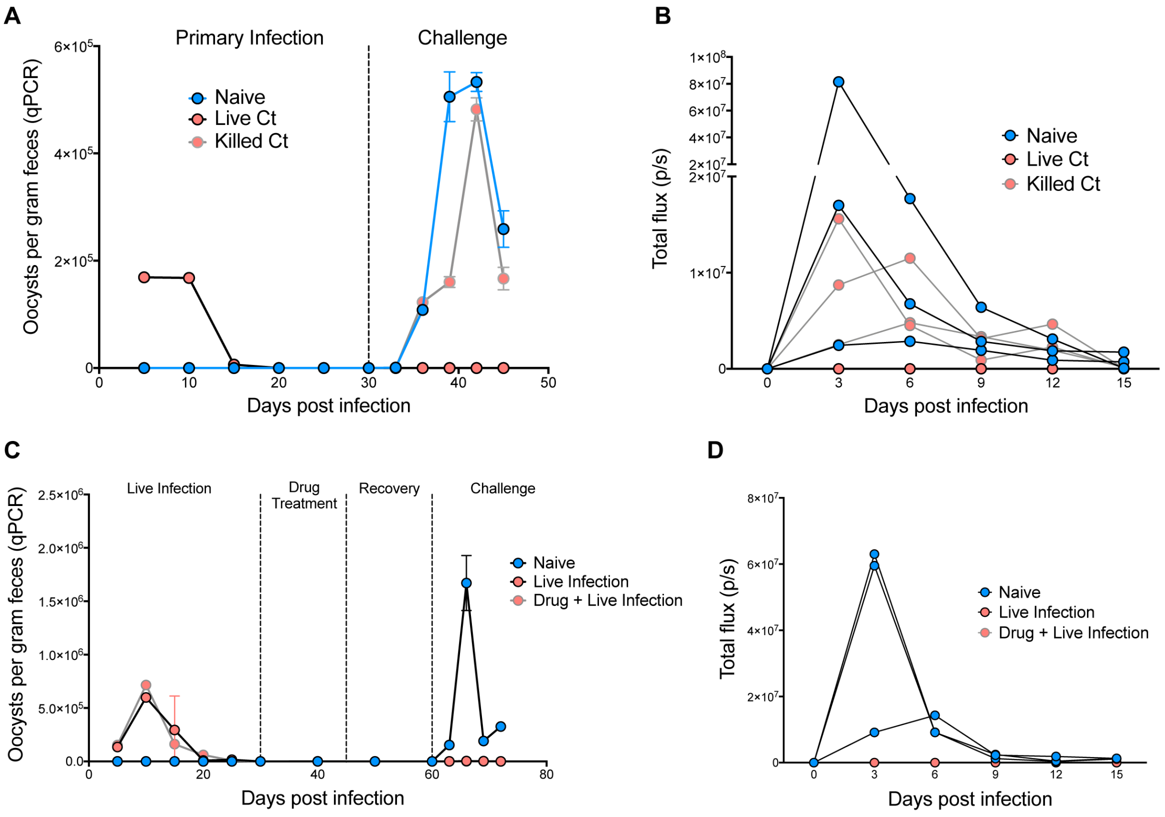

Figure S4: Fecal shedding and luminescent imaging of previously infected mice, related to Figure 6 (A-B) C57BL/6 mice were subjected to primary infection as indicated, followed by a challenge with 100,000 oocysts of transgenic *tyzzeri Ct*-LC. **(A)** Parasite shedding measured by qPCR of collected fecal samples throughout the primary infection and challenge. Two technical replicates and data shown is mean ± s.d.. **(B)** Parasite tissue burden measured by whole animal imaging following challenge. *n* = 9 mice, 3 per group. **(C-D)** Fecal shedding and luminescent imaging of drug treated mice. C57BL/6 mice were first subjected to primary infection with 10,000 live, wild-type, *C. tyzzeri* oocysts. Mice were then treated at day 30 with paromomycin to eradicate parasite infection (16mg/mL ad libitum) for 15 days and allowed to recover for an addition 15 days. At day 60 post infection, mice were challenged with 100,000 transgenic *Ct*-LC oocysts. **(C)** Parasite shedding measured by qPCR of collected fecal samples throughout the experiment. Two technical replicates and data shown is mean ± s.d.. **(D)** Parasite tissue burden measured by whole animal imaging following challenge. *n* = 9 mice, 3 per group.

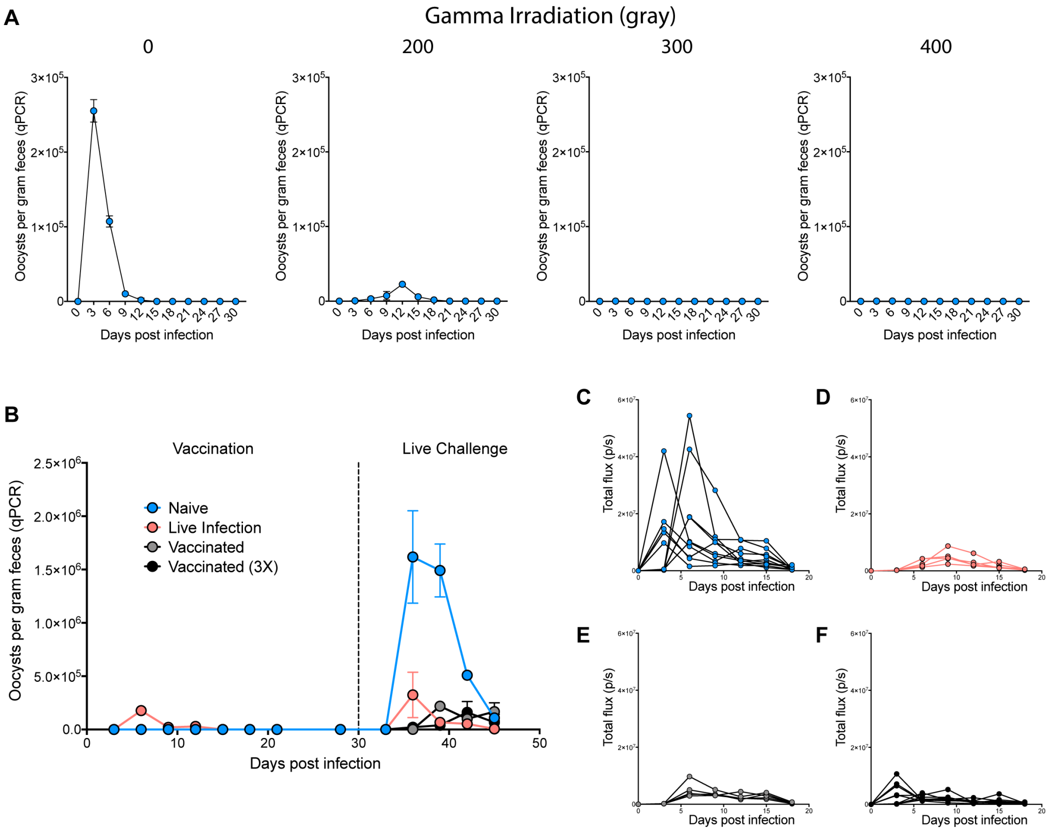

Figure S5: Attenuation of *Cryptosporidium tyzzeri* oocysts and fecal shedding and luminescent imaging of vaccinated mice, related to Figure 6 (A) Wild-type *C. tyzzeri* oocysts were exposed to the indicated dose of γ-irradiation using a Cs-137 or mock treatment, then mice were infected with 100,000 oocysts each and fecal shedding was monitored by qPCR. *n* = 12 mice, 3 per group, with qPCR performed on pooled fecal material with two technical replicates (data shown is mean ± s.d.). **(B-F)** Fecal shedding and luminescence imaging of vaccinated mice. C57BL/6 mice were first infected with 50,000 live or gamma irradiation attenuated (300gray) wild-type *C. tyzzeri* oocysts, then challenged with 100,000 live, transgenic, *Ct*-LC oocysts. **(B)** Parasite shedding measured by qPCR of collected fecal samples throughout the infection/vaccination and subsequent live challenge (data shown is mean ± s.d.). **(C-F)** Parasite tissue burden measured by whole animal imaging following the live challenge. *n* = 30 mice across 2 independent experiments, 10 Naïve, 5 Live Infection, 5 Vaccinated, and 10 Vaccinated (3X).

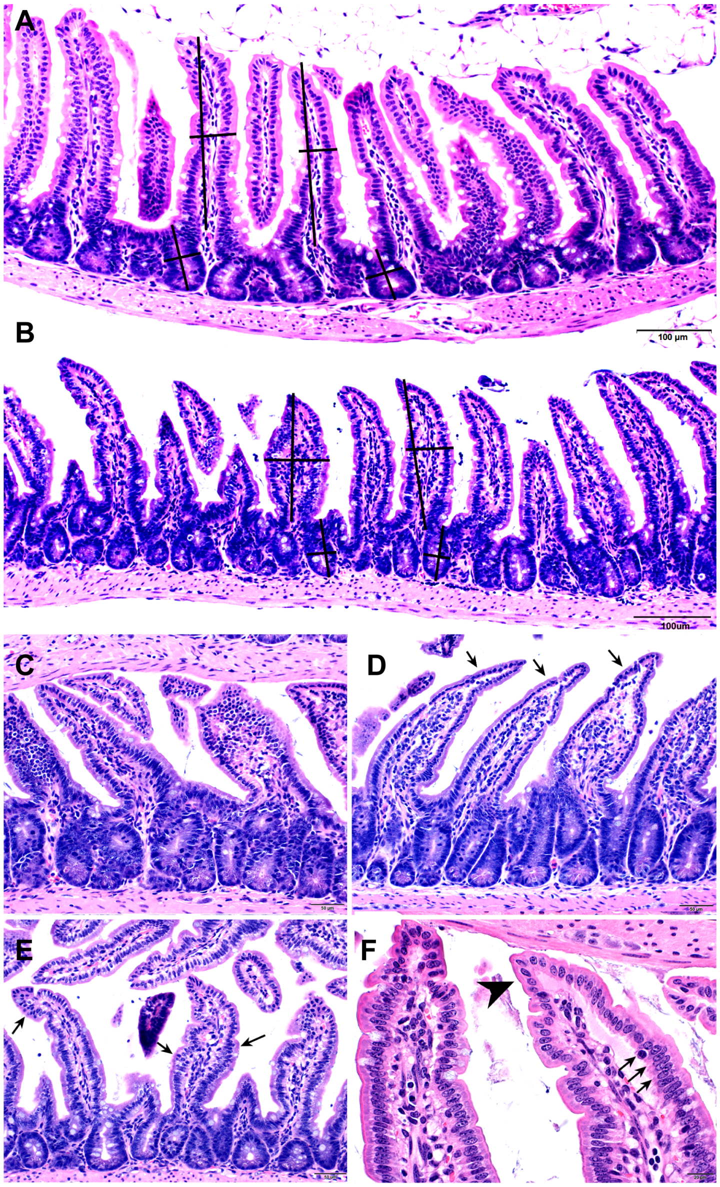

Figure S6: Select features of histologic scoring system, related to Star Methods and Figure 3 **(A-B)**, 10X magnification, H&E stained photomicrographs of distal jejunal segments from uninfected **(A)** and day 6 infected **(B)** mice depicting unlabeled measurement bars for villus height, mid-villus width, crypt height, and crypt width; scale bars = 100 µm. **(C-E)** 20X magnification, H&E stained photomicrographs of distal jejunal segments from day 6 infected mice; scale bars = 50 µm. **(C)** Greater than 2 crypt branches with distorted lumens affect multiple crypts within the section. Crypts also have an increased mitotic index when compared to uninfected mice. **(D)** Attenuated (cuboidal), hyperbasophilic epithelium having high nuclear:cytoplasmic ratios (**arrows**) line mid- to apical portions of multiple villi. **(E)** Villus epithelial dysplasia characterized by malignment of villus epithelial nuclei with cell piling (arrows). **(F)** 40X magnification, H&E stained photomicrograph. The villus to the right is lined

attenuated epithelium (**arrowhead**) with a cleft (**arrows**) filled with pale eosinophilic fluid separating the basal aspects of epithelium from the lamina propria; scale bar = 20 µm.
